# Integrating MaxEnt with chemometrics to evaluate the impact of environmental variables on the coumarin content and the distribution of *Angelica dahurica*

**DOI:** 10.3389/fpls.2025.1600491

**Published:** 2025-07-07

**Authors:** Zhengkun Gan, Jun Ma, Xinyu Liu, Jiaxin Luo, Junke Li, Lili Pu, Guihua Jiang, Yan Lian

**Affiliations:** ^1^ State Key Laboratory of Southwestern Chinese Medicine Resources, School of Pharmacy, Chengdu University of Traditional Chinese Medicine, Chengdu, China; ^2^ Xinjiang Uyghur Autonomous Region Academy of Surveying and Mapping, Urumqi, Xinjiang, China; ^3^ School of Ethnic Medicine, Chengdu University of Traditional Chinese Medicine, Chengdu, China

**Keywords:** *Angelica dahurica*, chemometrics, MAXENT model, HPLC, environmental variables, coumarin, multiple stepwise regression

## Abstract

**Introduction:**

*Angelica dahurica* is a traditional medicinal plant known for its high content of bioactive coumarins. With climate change potentially affecting both species distribution and secondary metabolite accumulation, there is a pressing need to integrate ecological and chemical data to guide future cultivation and resource utilization strategies.

**Methods:**

This study combined the Maximum Entropy (MaxEnt) ecological modeling approach with chemometric analysis to (i) predict the suitable habitat distribution of *A. dahurica* under current and future climate scenarios and (ii) evaluate the correlation between environmental variables and coumarin accumulation.

**Results:**

(1) The key environmental variables influencing the distribution of *A. dahurica* were identified as BIO_13 (precipitation of the wettest month), BIO_11 (mean temperature of the coldest quarter), and elevation (DEM). (2) Presently, the highly suitable regions for *A. dahurica* cultivation are mainly in Sichuan, Henan, and Hebei provinces. (3) Under future climate scenarios, the highly suitable habitats are expected to expand and shift geographically, especially toward Henan and Jiaozuo, with parts of Hubei, Shaanxi, and Shandong transitioning into highly suitable zones. (4) Chemometric analyses revealed that *A. dahurica* samples from highly suitable areas contained significantly higher total coumarin content than those from medium-suitability regions. (5) A strong correlation was observed between key environmental factors (especially BIO_11 and DEM) and the relative content of five major coumarin components.(6) Spatial mapping of chemical composition indicated distinct regional differences in coumarin distribution, suggesting the potential for geoherbalism-based classification.

**Discussion:**

The integration of ecological modeling with chemical analysis provides a powerful framework for understanding the impact of environmental variables on both the distribution and chemical quality of *A. dahurica*. These findings offer valuable guidance for targeted cultivation and resource management under future climate change conditions.

## 1 Introduction

**Figure 1:**
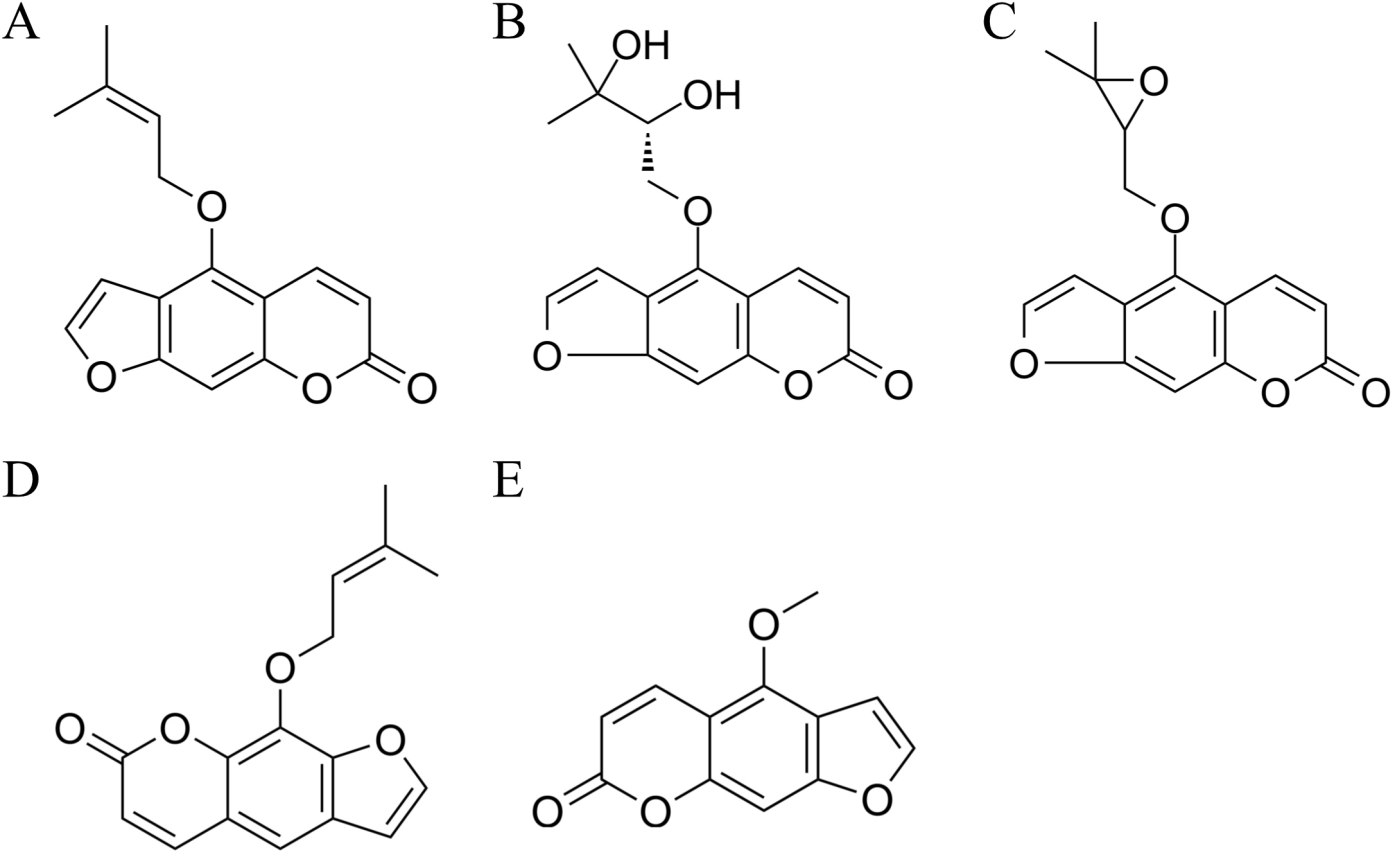
Chemical structures of the components detected in this study. **(A)** Isoimperatorin; **(B)** Oxypeucedanin hydrate; **(C)** Oxypeucedanin; **(D)** Imperatorin; **(E)** Bergapten.

*Angelica dahurica*, a member of the Apiaceae family, has dried roots that serve as key components in several traditional Chinese medicine prescriptions, such as Qingfeng Baidu San and Cang’erzi San State pharmacopoeia committee (2020). Modern pharmacological studies have demonstrated that *A. dahurica* possesses notable antibacterial, antioxidant, antitumor, and analgesic activities Zhao et al. (2022). Among the secondary metabolites of *A. dahurica*, coumarin serves as the primary active component. Coumarins are a class of benzopyrone derivatives known for their distinctive planar aromatic structures, which enable interactions with various biological targets. Structurally, they consist of a fused benzene and *α*-pyrone ring, with various substitutions influencing their pharmacological properties. For example, imperatorin and isoimperatorin possess furan rings, contributing to their lipophilicity and biological activity, including modulation of cytochrome P450 enzymes and anti-inflammatory pathways Dixon and Paiva (1995); Zhou et al. (2015). These compounds have attracted increasing interest due to their multi-target pharmacological activities. Recent reviews have emphasized that natural coumarins and isocoumarins exhibit significant anticancer potential by modulating apoptosis, autophagy, angiogenesis, drug resistance, and immune regulation pathways Aqib et al. (2025). Other studies have also pointed out their neuroprotective roles, especially in Alzheimer’s disease, where they can reduce A*β* aggregation, inhibit tau hyperphosphorylation, and regulate GSK-3 activity, offering a multitargeted therapeutic strategy Hussain et al. (2025). Furthermore, coumarins have been recognized as privileged scaffolds in medicinal chemistry. They demonstrate broad-spectrum activities, including anti-inflammatory, monoamine oxidase inhibition, antithrombotic, antidiabetic, hepatoprotective, and antimicrobial effects, underscoring their structural versatility and drug development potential Hussain et al. (2024). Additionally, the design of synthetic hybrids, such as benzocoumarin-stilbene derivatives, has shown promising antitumor efficacy through mechanisms like DNA ligase I inhibition, providing a potential lead for next-generation anticancer drugs Hussain et al. (2017). The chemical structures of the components detected in this study are shown in **Figure 1**.

*A. dahurica* plays a pivotal role in traditional Chinese medicine not only due to its rich coumarin content, but also because of its multifaceted therapeutic potential. The root contains bioactive components that exhibit antimicrobial, anti-inflammatory, neuroprotective, and hepatoprotective properties, contributing to its wide usage in treating skin diseases, rhinitis, headaches, and digestive disorders Zhao et al. (2022); Zhou et al. (2015). Additionally, its effects on the central nervous system and vascular systems make it biologically relevant in developing treatments for modern ailments such as migraines, atherosclerosis, and inflammation-related syndromes.

Various variables, including soil conditions, climate, and water sources, can influence the growth of *A. dahurica* and the accumulation of its medicinal constituents. For instance, the concentration of coumarin compounds in the roots of *A. dahurica* peaks in mid to late July. Additionally, different processing methods, such as sun drying and sulfur fumigation, impact the concentration of coumarin compounds, with sun drying being more effective in preserving these compounds Mengyue (2003). Since the Last Interglacial (LIG), the climate has undergone several drastic changes that have significantly affected the distribution of *A. dahurica* Blois et al. (2013). This species has a long history of cultivation in China, with records indicating its cultivation in Jiangsu and Zhejiang provinces as early as the late Ming Dynasty Wang et al. (2001). Currently, *A. dahurica* is produced in Hebei, Henan, Anhui, Sichuan, and other regions across China Ma et al. (2009), and it is also found in parts of Europe and North America Wang et al. (2020).

Global climate change poses a significant threat to the sustainable utilization of medicinal plants, primarily manifested through substantial reductions or shifts in their survival environments. Robiansyah et al. (2023). Cahyaningsih et al. Cahyaningsih et al. (2021) indicated that climate change has significantly impacted medicinal plants in Indonesia, highlighting the potential threat posed by global climate change to these vital species. The utilization of climate data to develop species distribution models has emerged as a widely adopted methodology among researchers studying the responses of various species to climate change Abdulwahab et al. (2022). The maximum entropy modeling (MaxEnt) is a widely used machine learning algorithm for predicting species distributions based on presence-only data. It estimates the probability distribution of maximum entropy (i.e., the most spread out or uniform distribution) constrained by environmental variables at known occurrence sites, thereby predicting suitable habitats in geographic space. MaxEnt has become a standard tool in ecological niche modeling due to its strong predictive performance, especially under limited or biased occurrence data conditions Baldwin (2009); Phillips et al. (2025). On the other hand, environmental variables significantly influence the accumulation of chemical components in medicinal plants Pant et al. (2021); Li et al. (2020). Recent studies have indicated that the habitat of medicinal plants is a critical factor in the accumulation of effective components Lu et al. (2020). Reports suggest that many plants found in highly suitable habitats exhibit elevated levels of chemical components Xu et al. (2018). Although the mechanism by which climate change affects plant quality remains unclear, the results of these studies may contribute to assessing the geographical distribution of future medicinal plants through habitat suitability analysis.

In addition, high-performance liquid chromatography (HPLC) can effectively evaluate the differences in chemical components of *A. dahurica* from different regions. Studies indicate that the quality of *A. dahurica* from Henan and Hebei is significantly superior to that from Sichuan and from Anhui Chen et al. (2019). However, these samples are predominantly concentrated in traditional production areas and their immediate surroundings. The quality of *A. dahurica* in other environments remains unclear, as does the impact of climate change on the distribution of highquality *A. dahurica*. In the face of the increasing demand for *A. dahurica* in the market in recent years, relevant industries and departments need to consider how to delineate suitable planting areas to achieve sustainable supply of *A. dahurica*. Currently, research on *A. dahurica* mainly focuses on its pharmacology and medicinal effects, and in the reported studies on the distribution of *A. dahurica*, although online database occurrence records have been used, the content of chemical components has not been included in the scope of investigation, and the evaluation of the quality of *A. dahurica* in areas that may be suitable for planting *A. dahurica* cannot provide comprehensive reference value Zhang et al. (2024a), which is not conducive to the formulation of planting area expansion plans.

This study aims to: (1) Predict changes in *A. dahurica*’s suitable habitat under climate change; (2) Identify key environmental variables affecting its distribution; (3) Analyze how habitat suitability influences coumarin accumulation.

## 2 Materials and methods

### 2.1 Samples and species occurrence records

**Figure 2:**
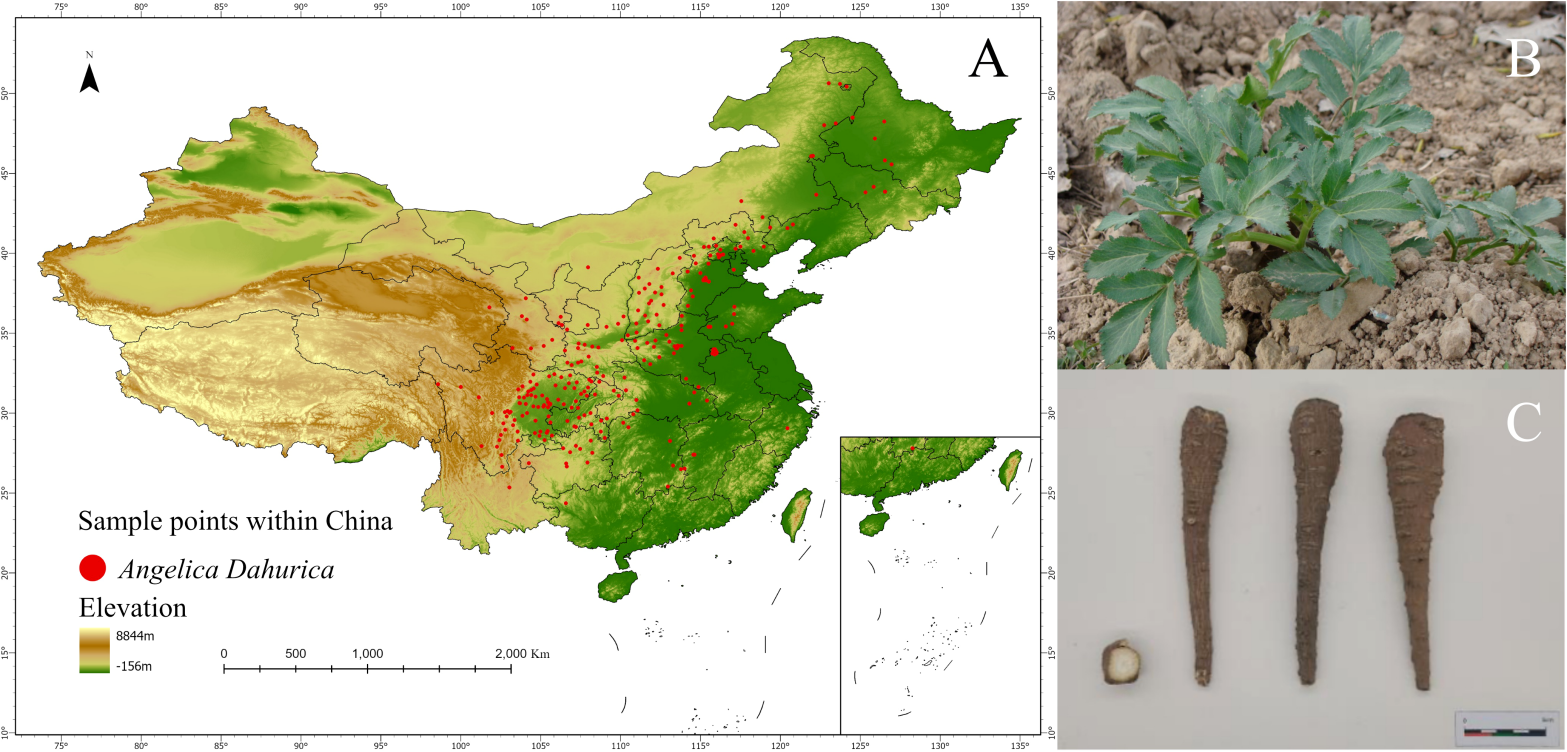
**(A)** Distribution of *A. dahurica*; **(B)** Original plant of *A. dahurica*; **(C)** Parts of *A. dahurica* used for medicine.

The species occurrence records of this study were obtained from the National Specimen Information Infrastructure(NSII) and the Chinese Virtual Herbarium(CVH). To prevent overfitting, ENMTools in R was used to filter occurrence data, retaining only one record per 1*km* ^2^ grid Wang et al. (2024b); Zhao et al. (2024). After screening, a total of 246 valid occurrence records were used in this study (**Supplementary Table S1** for details), as shown in **Figure 2**. And this study collected 28 batches of *A. dahurica* samples in Suining City, Jianyang City, Ziyang City, Luzhou City, and Guang’an City in Sichuan Province, Nanchuan District in Chongqing, Yuzhou City in Henan Province, Anguo City in Hebei Province, and Bozhou City in Anhui Province, with precise geographic coordinates recorded (**Table 1**). All 28 batches were taxonomically identified as *Angelica dahurica* (Fisch.ex Hoffm.) Benth.et Hook.f. or *Angelica dahurica* (Fisch.ex Hoffm.) Benth.et Hook.f.var.*formosana*(Boiss.) Shan et Yuan of the Apiaceae family by Professor Guihua Jiang of the School of Pharmacy, Chengdu University of Traditional Chinese Medicine.

**Table 1:** *A. dahurica* collection sites.

No.	Province	Local	Longitude	Latitude
S1	Hebei	Wurenqiao Town, Anguo	115.33	38.30
S2	Hebei	Dawunu Town, Anguo	115.22	38.37
S3	Hebei	Zhengzhang Town, Anguo	115.27	38.38
S4	Hebei	Xifo Township, Anguo	115.33	38.42
S5	Hebei	Beiduancun Township, Anguo	115.30	38.47
S6	Hebei	Ziwei Town, Dingzhou	115.19	38.31
S7	Anhui	Qiaodong Town, Bozhou	115.88	33.85
S8	Anhui	Shijiuli Town, Bozhou	115.84	33.80
S9	Anhui	Hua Tuo Town, Bozhou	115.78	33.94
S10	Anhui	Wuma Town, Bozhou	115.88	33.91
S11	Anhui	Shatu Town, Bozhou	115.96	33.72
S12	Anhui	Yanji Town, Bozhou	115.89	33.98
S13	Anhui	Zhangdian Township, Bozhou	115.93	33.93
S14	Anhui	Guantang Town, Bozhou	116.02	33.80
S15	Sichuan	Shuangyan Township, Yuechi County, Guang’an	106.44	30.54
S16	Sichuan	Yongxing Town, Chuanshan District, Suining	105.61	30.57
S17	Sichuan	Sanquan Town, Nanchuan District, Chongqing	107.21	29.13
S18	Sichuan	Tang’s hometown in Chuanshan District, Suining	105.51	30.63
S19	Sichuan	Lianhua Township, Anju District, Suining	105.46	30.36
S20	Sichuan	Xinqiao Town, Chuanshan District, Suining	105.53	30.56
S21	Sichuan	Taixing Township, Shehong County, Suining	105.59	30.53
S22	Sichuan	Li Jia Town, Anyue County, Ziyang	105.48	29.80
S23	Sichuan	Quba Town, Naxi District, Luzhou	105.37	28.77
S24	Sichuan	Pingping Township, Jianyang, Chengdu	104.55	30.41
S25	Sichuan	Yongquan Town, Jianyang, Chengdu	104.85	30.38
S26	Henan	Xiaolu Township, Yuzhou	113.48	34.05
S27	Henan	Guolian Township, Yuzhou	113.54	34.07
S28	Henan	Gucheng Town, Yuzhou	113.56	34.23

### 2.2 Environmental variables

Species distribution is influenced by both abiotic variables (e.g., climatic conditions, edaphic properties, and topographic features) and biotic components. For modeling the current distribution pattern of *A. dahurica*, 31 candidate environmental variables were selected, including: Bioclimatic variables for the current period (1970-2000), 2041-2060, and 2061–2080 provided by the World Climate Database (www.worldclim.org) under the assumption of future carbon emissions as SSP126 (the most stringent carbon emission control scenario) Yang et al. (2025); Soil-related variables provided by the Harmonized World Soil Database (HWSD, version 1.1) constructed by the Food and Agriculture Organization (FAO) and the International Institute for Applied Systems Analysis (IIASA) Lu and Liu (2019): ADD_PROP [Other properties (gelic, vertic, petric)], AWC_CLASS (AWC Range), REF_DEPTH (Reference Soil Depth), SU_SYM90 [Soil Unit Symbol (FAO-90)], T_CACO3 (Topsoil Calcium Carbonate), T_CEC_CLAY [Topsoil CEC (clay)], T_CEC_SOIL [Topsoil CEC (soil)], T_GRAVEL (Topsoil Gravel Content), T_OC (Topsoil Organic Carbon), T_PH_H2O [Topsoil pH (H2O)], T_SAND (Topsoil Sand Fraction), T_SILT (Topsoil Silt Fraction), T_TEXTURE (Topsoil Texture), and other soil data; DEM (Elevation) provided by the General Bathymetric Chart of the Oceans (GEBCO, www.gebco.net) GEBCO Bathymetric Compilation Group (2023). The spatial resolution of these data is 30 seconds (≈ 1*km*).

Excessive environmental variables can result in overfitting issues, such as multicollinearity and autocorrelation within the model Yang et al. (2025). This study identified environmental variables with non-zero contribution rates through preliminary experiments. Subsequently, Pearson correlation analysis was conducted using R to evaluate the selected environmental variables, leading to the selection of those with a correlation coefficient |*r*| ≥ 0.8 and a significant contribution rate (See **Supplementary Table S2** for relevant analysis). Ultimately, the model retained four climatic variables (BIO_13, BIO_11, BIO_19, BIO_15), three soil variables (T_PH_H2O, REF_DEPTH, T_CEC_CLAY), and one topographic variable (DEM) for predicting the potential suitable habitat of *A. dahurica*, as illustrated in **Table 2**.

**Table 2:** Variables used in MaxEnt and their contribution, permutation importance.

Variable	Description	Contribution (%)	Permutation importance (%)	UNITS
BIO_13	Precipitation of wettest month	34.6	22.7	mm
BIO_11	Mean temperature of coldest quarter	24.4	41.5	°C
DEM	Elevation	13.3	13.4	m
T_PH_H2O	Topsoil pH (H2O)	8.4	0.9	-log(*H* ^+^)
REF_DEPTH	Reference Soil Depth	8.2	2.9	m
BIO_19	Precipitation of coldest quarter	6.6	15.4	mm
T_CEC_CLAY	Topsoil CEC (clay)	3.5	0.9	cmol/kg
BIO_15	Precipitation seasonality	0.9	2.2	mm

### 2.3 Species distribution modeling process

#### 2.3.1 Model establishment and optimization

MaxEnt is widely used to predict potential species distribution areas. It correlates species occurrence data with environmental variables using a specific algorithm, then applies this relationship across the study area to generate distribution predictions Zhao et al. (2024). The R package ENMeval optimizes MaxEnt models by reducing complexity and overfitting, improving prediction accuracy Kass et al. (2021).

In this study, ENMeval was used to optimize MaxEnt parameters. The Regularization Multiplier (RM) values (0.5–5, step 0.5) were tested with five feature types (L, LQ, H, LQH, LQHP, LQHPT), forming 60 combinations. Using 246 *A. dahurica* occurrence points and eight environmental variables, ENMeval performed cross-validation and optimization under different RM and feature class (FC) combinations Wang et al. (2023, 2024a). Model selection was based on the Akaike Information Criterion (AICc) for complexity and fit, while the 10% training omission rate (avg.OR10) and the average AUC difference (avg.diff.AUC) assessed model performance. The model with the lowest deltaAICc was chosen as the optimal model Lin et al. (2023).

#### 2.3.2 Predictive results and evaluation of the model

In this study, MaxEnt (Version 3.4.4) Phillips et al. (2025) was used to analyze and predict suitable habitats for *A. dahurica*, 75% of the occurrence data was used for training and 25% for testing. The model was run with 10 Bootstrap replicates Zhao et al. (2021b). Model performance was evaluated using the Area Under the Curve (AUC) of the Receiver Operating Characteristic (ROC) curve. AUC values range from 0 to 1, with higher values indicating greater predictive accuracy: <0.7 (poor), 0.7–0.8 (moderate), 0.8–0.9 (good), and >0.9 (excellent) Zheng et al. (2024); Zhan et al. (2022); Wang et al. (2024a); Qiu et al. (2022).

#### 2.3.3 Suitable habitat division of *A. dahurica*

In this study, ArcGIS Pro (Version 3.0.2) was used to classify the habitat suitability of *A. dahurica* using the natural breakpoint method Zhao et al. (2024); Zheng et al. (2024); Zhao et al. (2021b). Suitability was categorized into four levels: high, medium, low, and unsuitable habitat. The jackknife method was used to assess the relative influence of environmental variables. The key variables affecting *A. dahurica* distribution and their suitable ranges were identified based on variable contribution rates and permutation importance Wang et al. (2024a).

### 2.4 Changes in the core distribution

ArcGIS Pro was used to analyze changes in *A. dahurica*’s suitable habitat over time. Regional data from three periods were simplified into vector cores, and the change trend of the suitable area was calculated. Centroid shifts were used to indicate the migration direction of suitable habitats Qiu et al. (2022); Zhan et al. (2022).

### 2.5 Materials treatment and HPLC analysis

28 batches of *A. dahurica* samples were washed, dried, and ground. Approximately 1g of powder was accurately weighed into a 50mL conical flask, followed by the addition of 25mL methanol. After weighing, the sample was sonicated (300W, 40kHz) for 1h, removed and cooled, the weight was adjusted, and the filtrate was collected after shaking and filtering. The resulting filtrate was passed through a 0.22*µ*m microporous membrane and collected for further analysis. Standard solutions of imperatorin, imperatorin oxide, isoimperatorin, hydrated imperatorin oxide, and bergapten were prepared by dissolving accurately weighed amounts in methanol to achieve final concentrations of 0.1028 mg/mL, 0.1239 mg/mL, 0.0519 mg/mL, 0.0258 mg/mL, and 0.00968 mg/mL, respectively.

In this study, the contents of five coumarins were determined using HPLC on an Agilengt 1200 Infinity LC high performance liquid chromatograph (Agilent) and WondaSil C1 8-WR (250×4.6*mm*) chromatographic column (Shimadzu Jiyou Trading Co., Ltd.). The mobile phase was methanol (A), tetrahydrofuran (B) and pure water (C), gradient elution (0 ∼ 10 min, 32% A, 8% B;10 ∼ 45 min, 32% ∼ 53% A, 8% ∼ 12% B; 45 ∼ 55 min, 53% A, 12% B; 55 ∼ 65 min, 53% ∼ 32% A, 12% ∼ 8% B), flow rate was 1.0 ml/min, detection wavelength 305 nm, column temperature 25°C, injection volume 10*µ*l. By comparing the retention time wit standards, the isolated compounds were identified, and quantitative analysis was performed using peak area measurements.

### 2.6 HCA analysis

HCA (Hierarchical Cluster Analysis) is a commonly used unsupervised clustering analysis method, which divides samples into different clusters by calculating the similarity between samples. The ward method clusters by minimizing the variance within each cluster. In this study, the content data of the five coumarins measured in HPLC were standardized, and the ward method was used for cluster analysis and the dendrogram of the results was drawn using a Python program.

### 2.7 Construct a model of the relationship between content and environmental variables

Multiple regression analysis is a statistical method for modeling the relationship between a dependent variable and multiple independent variables. Stepwise regression refines this process by systematically adding or removing variables to identify the most significant predictors and construct the best predictive model. In this study, ArcGIS Pro was used to extract eight environmental variables from the coordinates of 28 *A. dahurica* samples. R was then used to perform multiple stepwise regression analysis, correlating environmental variables with the measured content of imperatorin, imperatorin oxide, isoimperatorin, hydrated imperatorin oxide, and bergapten in *A. dahurica*. This analysis established a relationship model between chemical composition and environmental variables. Using raster calculation and mapping functions in ArcGIS Pro, unsuitable areas from the ecological suitability prediction were removed. The spatial distribution of the five coumarins in *A. dahurica* under current and future climate conditions was then predicted and visualized.

## 3 Results

### 3.1 Model evaluation

**Figure 3:**
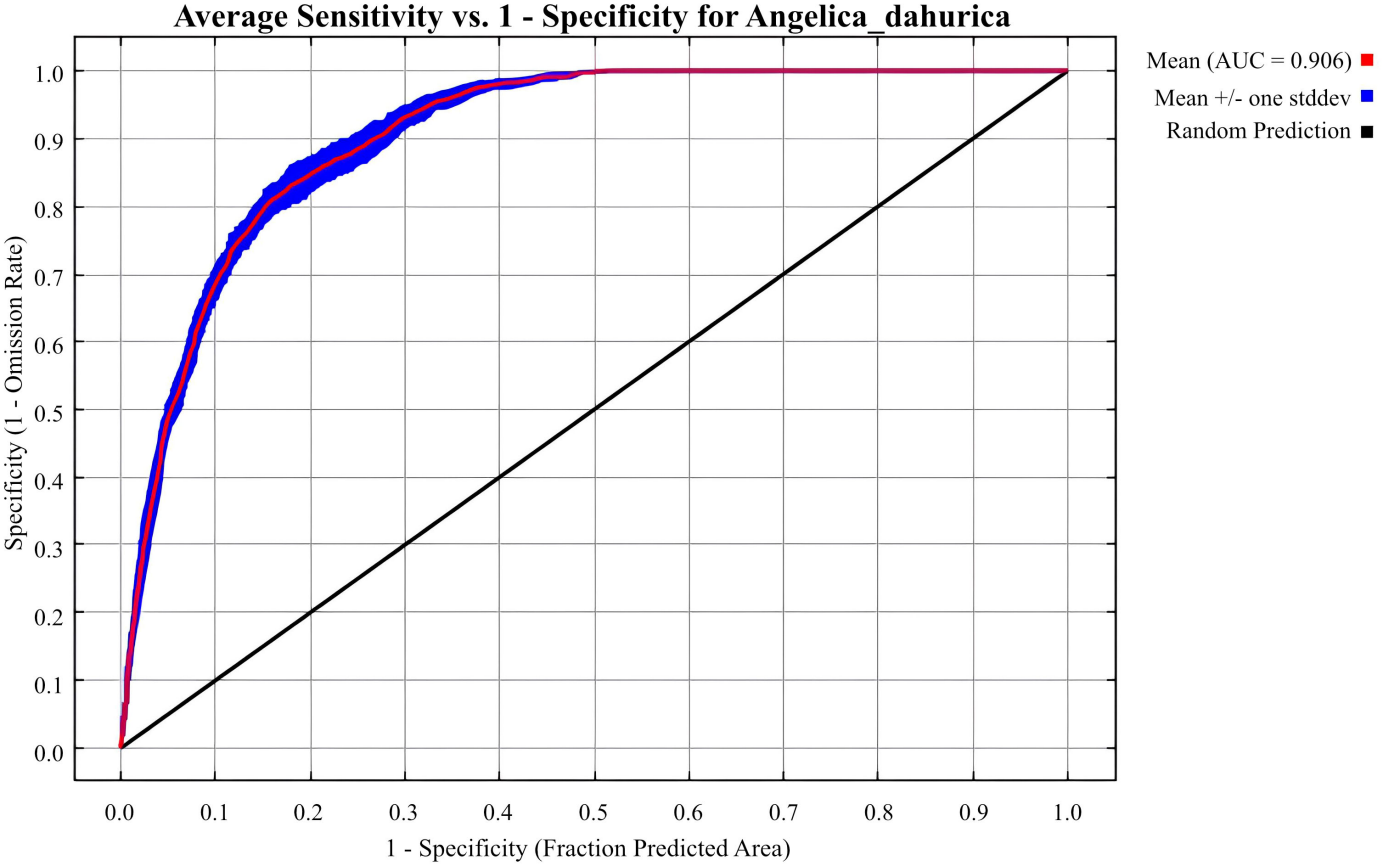
The receiver operating characteristic (ROC) curve for *Angelica dahurica*.

The default FC in MaxEnt is LQHPT with a RM of 1, yielding a delta.AICc of 28.18 and an Auc.Diff.Avg of 0.01956145. When FC is set to LQH and RM to 2, delta.AICc is 0, and Auc.Diff.Avg is 0.01983218, indicating an optimal model (**Table 3**). The average training AUC for the replicate runs(10 repetitions) is 0.906, and the standard deviation is 0.006 (**Figure 3**), confirming the model’s predictive accuracy. Compared to the default model, the response curve of the optimized model, adjusted using the ENMeval package, is significantly smoother and aligns more closely with a normal distribution, consistent with Shelford’s tolerance law Shelford (1931); Zhao et al. (2021a).

**Table 3:** Accuracy evaluation of MaxEnt with different parameters.

Type	FC	RM	Auc.Diff.Avg	OR10	delta.AICc
default	LQHPT	1	0.01956145	0.13478836	28.188223
optimized	LQH	2	0.01983218	0.12010582	0

### 3.2 Analysis of environmental variables

**Figure 4:**
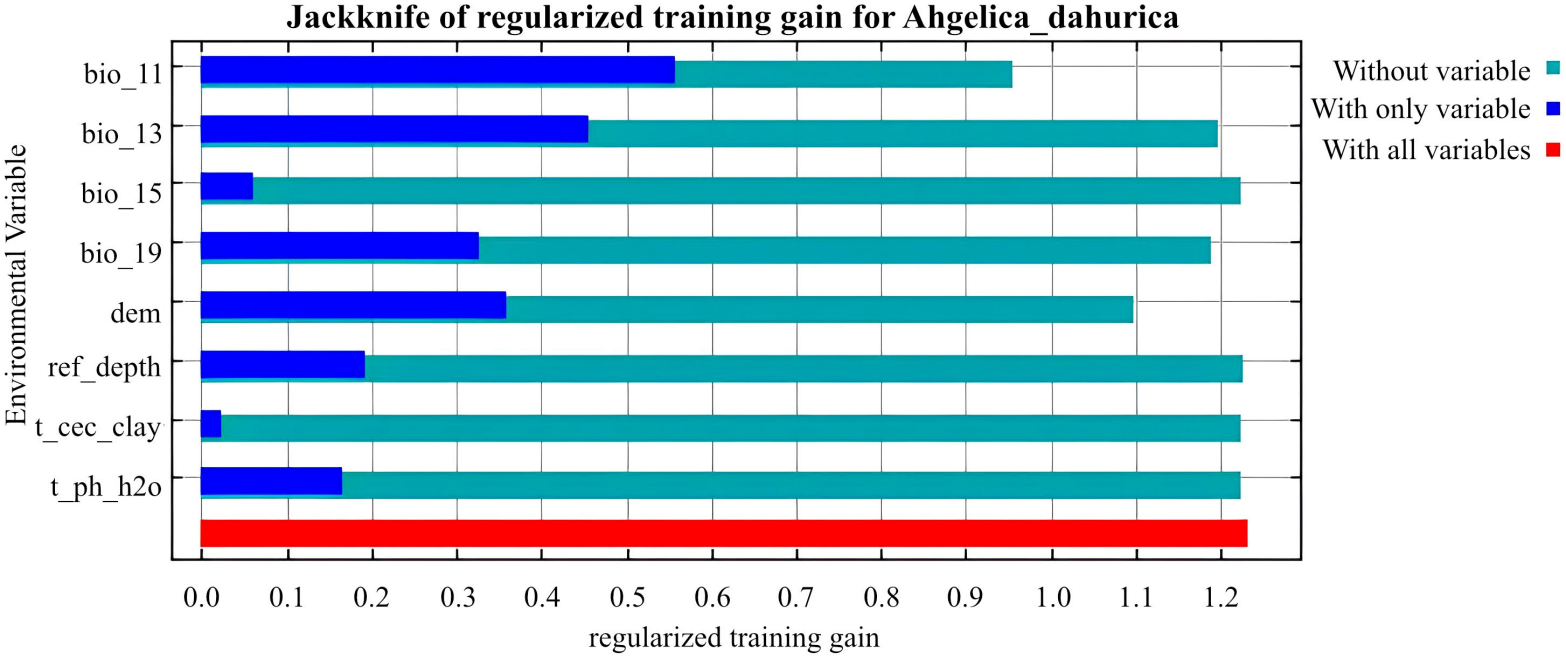
Jackknife of regularized training gain for *A. dahurica*.

**Figure 5:**
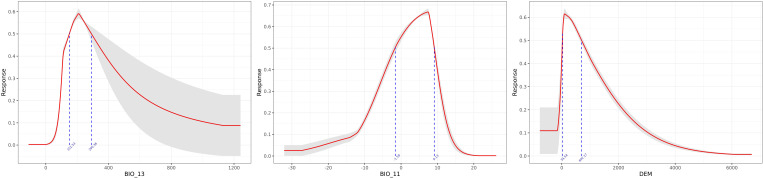
The response curves of the three most important environmental variables.

As shown in **Table 2**, BIO_13, BIO_11, and DEM are the key environmental variables influencing the distribution of *A. dahurica*. Their cumulative contribution rate and permutation importance reach 72.3% and 77.6%, respectively. BIO_13 has the highest contribution rate, while BIO_11 has the greatest permutation importance. When analyzed individually, temperature and precipitation related variables (such as BIO_13 and BIO_11) carry the highest weights (**Figure 4**). As shown in **Figure 5**, the three response curves follow a unimodal distribution.

### 3.3 Prediction of distribution under current climate situation

**Figure 6:**
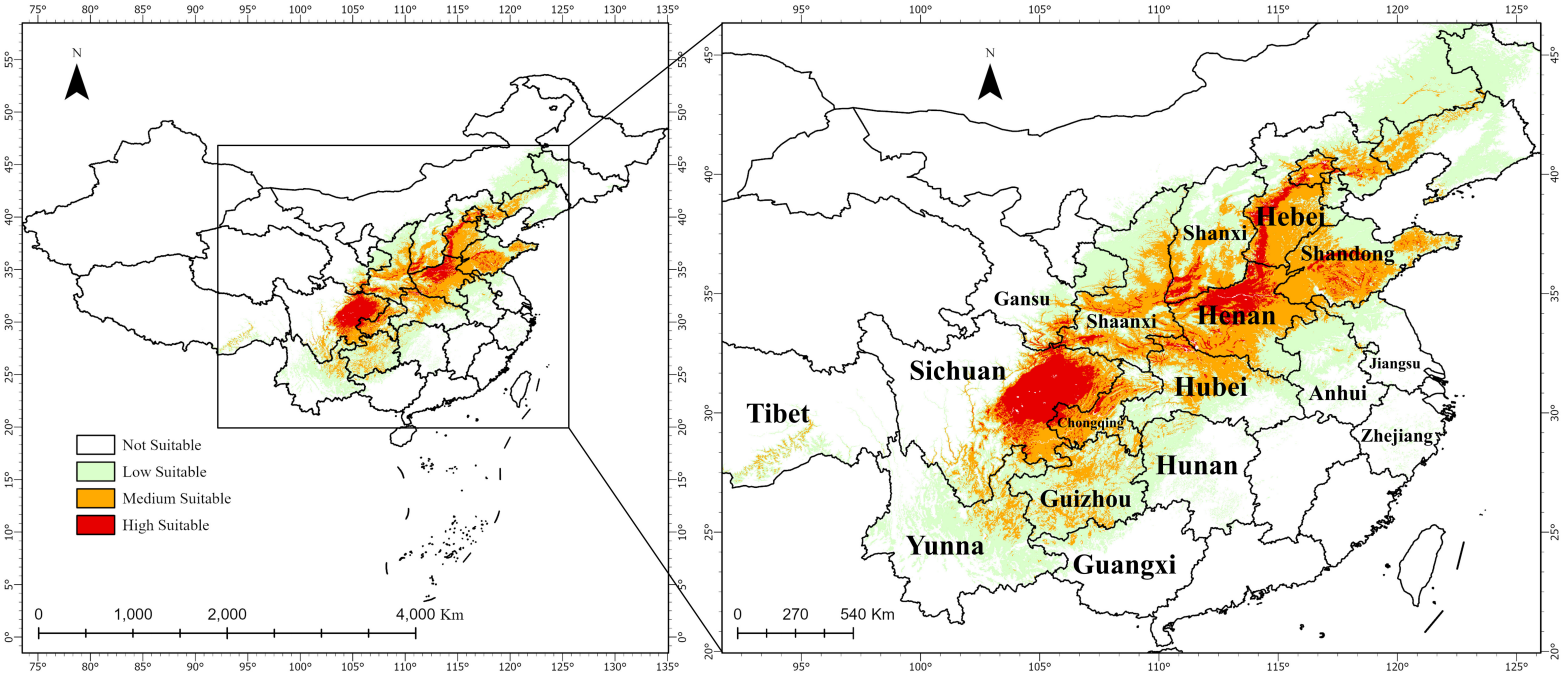
Distribution of suitable habitats for *A. dahurica* in China under current climate scenarios.

The results indicate that the average suitability of the 246 distribution records is 0.530025, with a maximum of 0.857906 (Shehong City, Suining, Sichuan Province) and a minimum of 0.052913 (Hulunbuir, Inner Mongolia). Under the current climate scenario, the total suitable habitat area (highly and mediumly suitable) is 89.11020 × 104 km2 (**Figures 6A**), with highly suitable areas covering 19.54812 × 10^4^ *km* ^2^. These are primarily located in Guangyuan, Bazhong, Dazhou, Nanchong, Suining, and Mianyang (Sichuan); Luoyang, Jiaozuo, Zhengzhou, Hebi, and Anyang (Henan); and Handan, Shijiazhuang, and Baoding (Hebei). Mediumly suitable habitats span 69.56208 × 10^4^ *km* ^2^, surrounding highly suitable regions, including western Chongqing, central and southern Henan, and western Shandong (**Figure 6B**).

### 3.4 Predict the future distribution of *A. dahurica*

#### 3.4.1 Future changes in suitable habitat for *A. dahurica*

**Figure 7:**
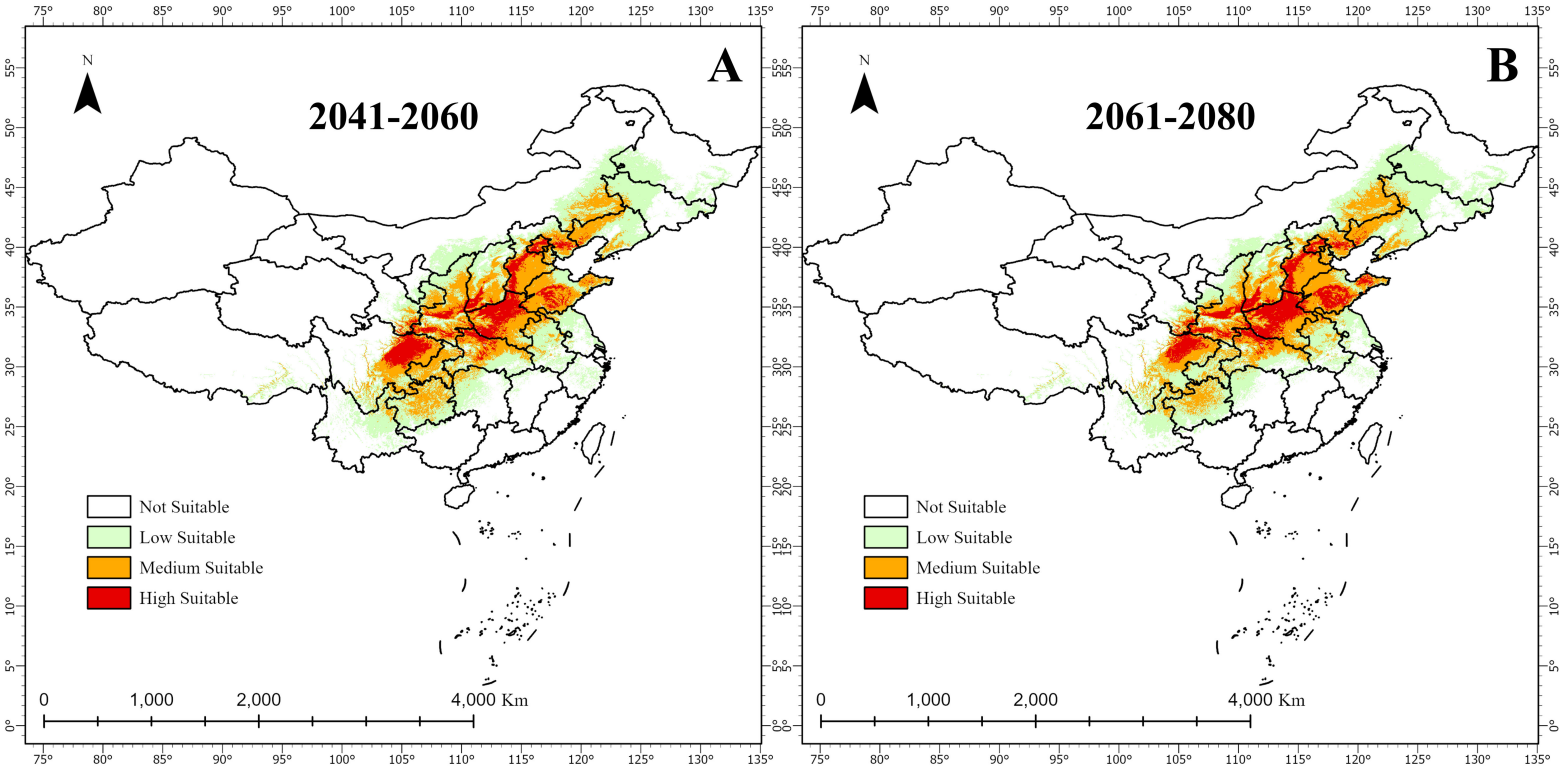
Future changes in suitable habitats for *A. dahurica* in China; **(A)** 2041-2060; **(B)** 20612080.

**Figure 7** and **Table 4** illustrate future changes in *A. dahurica*’s suitable habitats. Under different climate scenarios, highly, mediumly, and lowly suitable areas will expand or contract to varying degrees. Compared to the current climate: Under the 2041–2060 scenario, highly, mediumly, and lowly suitable habitats will increase by 8.50875 × 10^4^ *km* ^2^, 21.95549 × 10^4^ *km* ^2^, and 13.0805 × 10^4^ *km* ^2^, respectively; Under the 2061–2080 scenario, these areas will increase by 13.01056 × 10^4^ *km* ^2^, 18.05604×10^4^ *km* ^2^, and 7.6497×10^4^ *km* ^2^, respectively (**Table 4**). Overall, highly suitable habitats are projected to expand over time, allowing *A. dahurica* to grow and reproduce across a wider range, increasing its population and distribution. While medium and low suitability areas fluctuate, some regions in northern Hubei, central and southern Shaanxi, and Shandong may transition into highly suitable habitats.

**Table 4:** Area of suitable areas for *A. dahurica* under different climate scenarios.

Group	Current(×10^4^ *km* ^2^)	2041-2060(×10^4^ *km* ^2^)	2061-2080(×10^4^ *km* ^2^)
Not suitability area	725.9551	682.4103	687.2387
Low suitability area	123.5592	136.6397	131.2089
Medium suitability area	69.56208	91.51757	87.61812
High suitability area	19.548125	28.05687	32.55868

#### 3.4.2 Changes in core locations of suitable habitat

Currently, the core of the suitable habitat for *A. dahurica* is located in Yunxi County, Shiyan City, Hubei Province (110.54221, 33.209579). It is predicted that under the 2041–2060 climate scenario, the core of the suitable habitat will migrate to Yichuan County, Luoyang City, Henan Province (112.349646, 34.386318), migration distance is 211.78 km, and under the 2061–2080 climate scenario, it will migrate to Wen County, Jiaozuo City, Henan Province (113.01311, 34.884234), migration distance is 82.32 km, as shown in **Supplementary Figure S1**.

### 3.5 Analysis of coumarin content by HPLC

**Figure 8:**
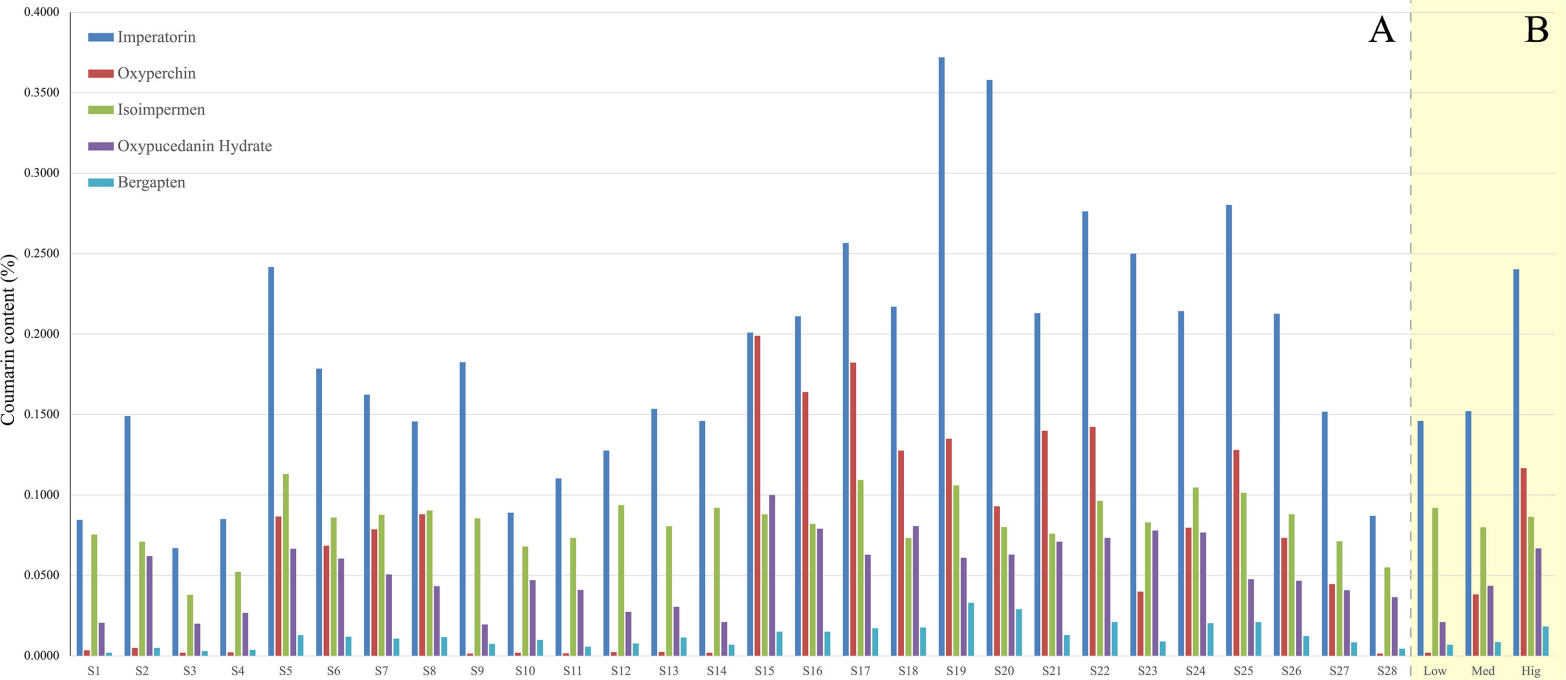
**(A)** The coumarin content of *A. dahurica* in each sample; **(B)** Average coumarin content of *A. dahurica* in different suitable areas (Low, low suitable area; Med, median suitable area; Hig, high suitable area).

HPLC results show variability in the five coumarin contents across the 28 sample batches (**Figure 8A**). Except for S3 from the mediumly suitable area, imperatorin content in all other samples (**Figure 8A**; dark blue) meets the PPRC standard (≥ 0.080%)State pharmacopoeia committee (2020). Apart from isoimpermen (**Figure 8B**; green), the average content of the other four coumarins is higher in samples from highly suitable areas compared to those from moderate and low suitability regions. This suggests that *A. dahurica* grown in highly suitable areas has superior quality. Additionally, isoimpermen content does not show a consistent increase with habitat suitability.

### 3.6 Analysis of HCA

**Figure 9:**
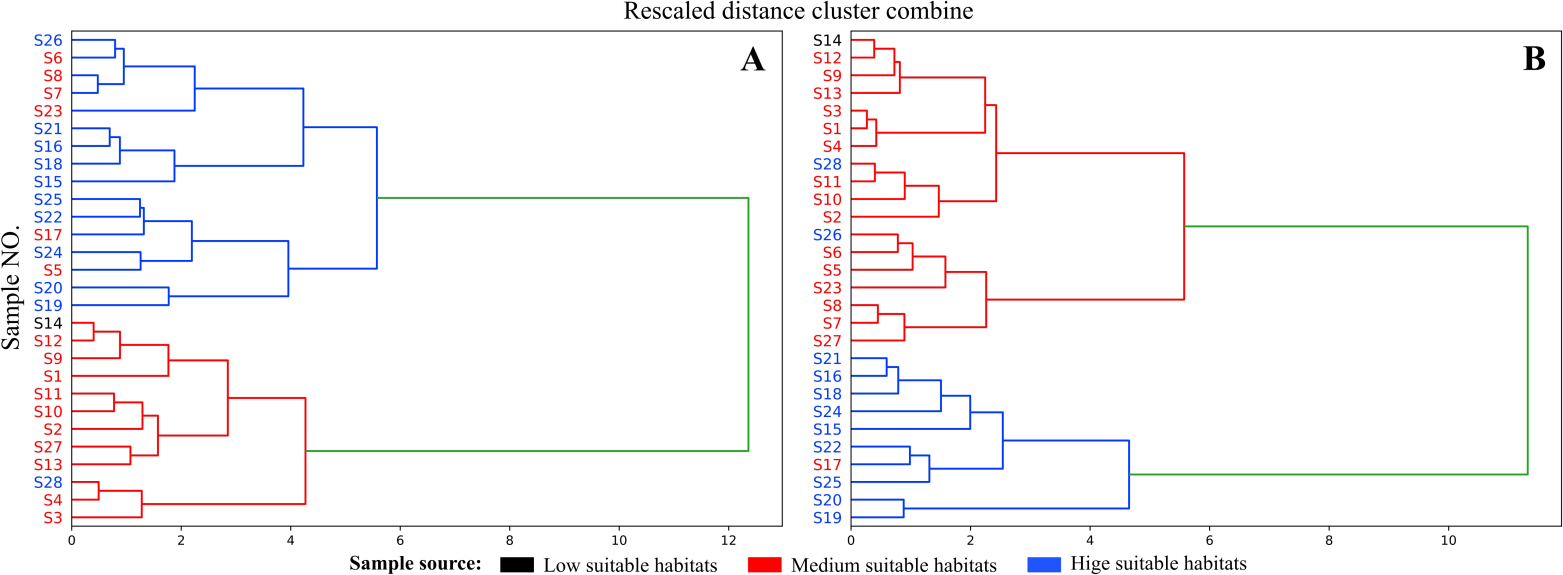
**(A)** Cluster results obtained using five coumarin contents; **(B)** Cluster results obtained after removing Isomperatorin.

The HCA results (**Figure 9**) show that when the critical value approaches 6, the 28 sample batches are divided into two distinct clusters. Samples S5, S6, S7, S8, S17, and S23 from the mediumly suitable area cluster with those from the highly suitable area (**Figure 9A**; blue), while the remaining samples form a separate cluster. However, **Figure 8B** (green) reveals that isoimpermen content does not correlate with habitat suitability, suggesting it may influence clustering results. After removing isoimpermen data and reanalyzing, the clustering improved: at a critical value of 6, only three samples (S17, S26, S28) were misclassified (**Figure 9B**). This revised classification more accurately reflects the actual distribution, with highly suitable area samples forming one cluster and moderately or low suitable area samples forming another.

### 3.7 Analysis of the relationship between environmental variables and contents of chemical components

HPLC data and the 8 environmental variables used in MaxEnt were analyzed using multiple stepwise regression to establish relationship models between chemical components and environmental variables (**Equations 1**–**5**). Each model has a P-value < 0.001, indicating strong statistical significance and suitability for predicting component content. The models show that: Imperatorin (**Equation 1**) is primarily influenced by BIO_11, BIO_13, and T_PH_H2O; Oxypeucedanin (**Equation 2**) is affected by BIO_11, BIO_19, DEM, and T_CEC_CLAY; Isoimperatorin (**Equation 3**) is influenced by BIO_11, BIO_19, DEM, and T_PH_H2O; Oxypeucedanin hydrate (**Equation 4**) is mainly affected by BIO_11, BIO_13, BIO_15, and T_CEC_CLAY; Bergapten (**Equation 5**) is influenced by BIO_11, BIO_15, DEM, and T_PH_H2O. Notably, BIO_11 affects all five coumarins, while DEM influences multiple components, highlighting their key roles in *A. dahurica* metabolite production.


(1) $$\begin{array}{l} Imperatorin=0.1886222+0.0293253\times BIO\_11-0.0016331\times BIO\_13+0.0282338\times T\_PH\_H2O\\ P=4.87E-06 \end{array}$$

(2) $$\begin{array}{l} \begin{array}{c} Oxypeucedanin=-0.03848+0.00895781\times BIO\_11-0.001261\times BIO\_19+0.0002886\times DEM+0.001159\times T\_CEC\_CLAY\; \end{array}\\ P=7.75E-09 \end{array}$$

(3) $$\begin{array}{l} Isoimperatorin=0.004783\;-0.003535\times BIO\_11+0.000254\times BIO\_19+0.0009924\times DEM+0.008861\times T\_PH\_H2O\\ P=0.001498 \end{array}$$

(4) $$\begin{array}{l} \begin{array}{l} Oxypeucedanin\;hydrate=0.0330589+0.0092975\;\times BIO\_11-0.0003382\times BIO\_13+0.000232\times BIO\_15+0.0004243\times T\_CEC\_CLAY\; \end{array}\\ P=3.40E-07 \end{array}$$

(5) $$\begin{array}{l} \begin{array}{c} Bergapten=-0.01797+0.0001567\times BIO\_11+0.00003667\times BIO\_15+0.0002685\times DEM+0.002857\times T\_PH\_H2O \end{array}\\ P=5.12E-08 \end{array}$$

### 3.8 Spatial distribution of relative content of chemical components

**Figure 10:**
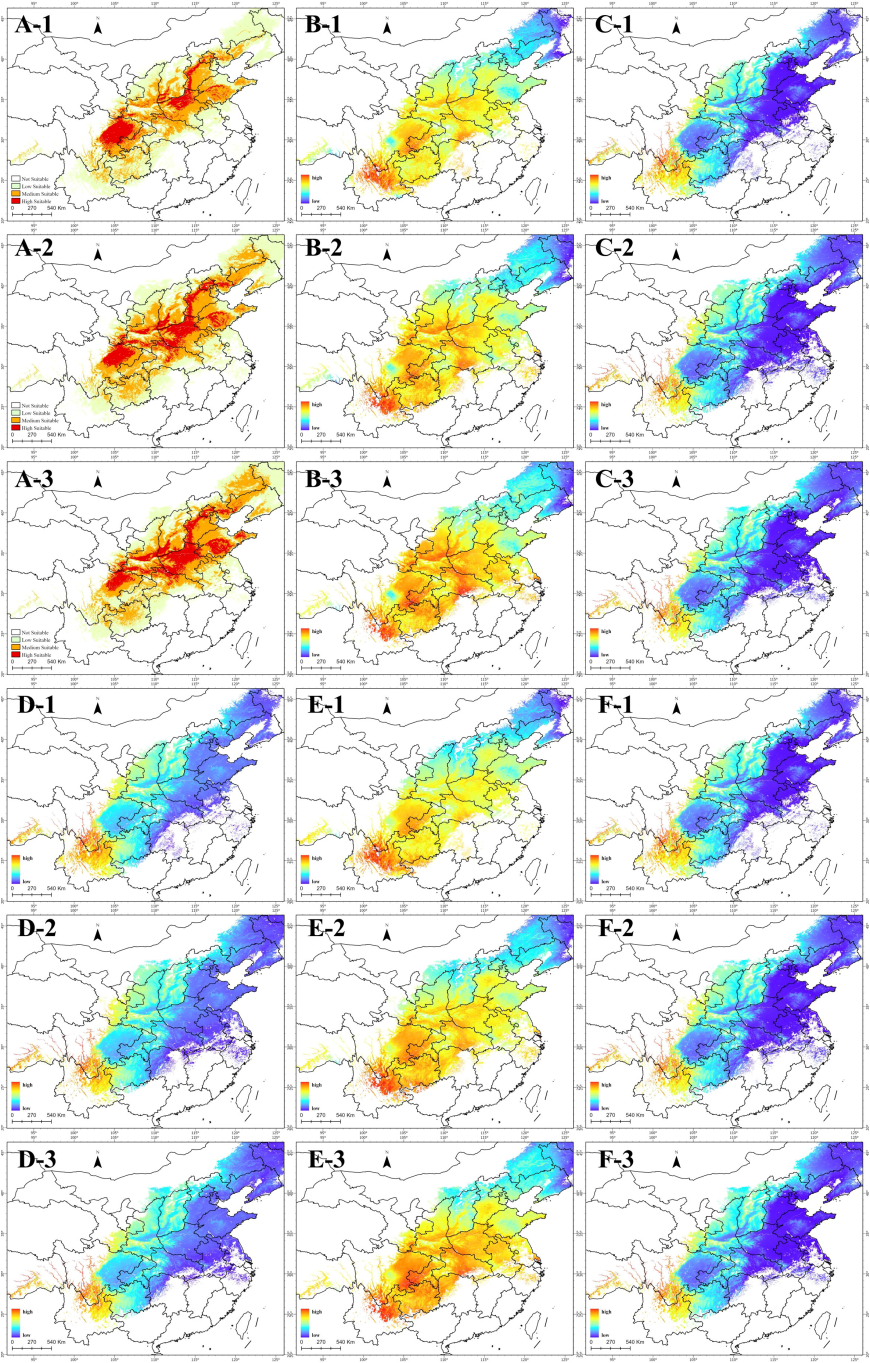
**(A)** Suitable area; **(B)** Relative content of Imperatorin; **(C)** Relative content of Isoimpermen; **(D)** Relative content of Oxyperchin; **(E)** Relative content of Oxypucedanin Hydrate; **(F)** Relative content of Bergapten; 1, current; 2, 2041-2060; 3, 2061-2080.

Based on the relationship model between content and environmental variables, obtain spatial distribution maps of the relative content of various coumarin components in suitable habitats areas at different periods. As shown in **Figure 10**, coumarin content in highly suitable habitats differs significantly from that in other suitable areas. Comparing the distribution maps (**Figure 10A**) reveals that imperatorin (**Figure 10B**) and oxypucedanin hydrate (**Figure 10E**) are more abundant in highly suitable areas, while isoimpermen (**Figure 10C**), oxyperchin (**Figure 10D**), and bergapten (**Figure 10F**) are less concentrated. Additionally, all five coumarins share a common trend: higher relative content in high-altitude regions, particularly at the junction of Yunnan, Guizhou, and Sichuan.

## 4 Discussion

### 4.1 Key environmental variables affecting *A. dahurica* distribution

*A. dahurica* is highly adaptable, thriving in mild, humid climates with ample sunlight. It has strong cold resistance but does not tolerate high temperatures. Based on response curves with values exceeding 0.5, several key environmental thresholds were identified: (1) The value range of BIO_13 is 152.53 ∼ 290.36*mm*, indicating that *A. dahurica* more suitable for growth in environments with concentrated seasonal precipitation, which is in line with *A. dahurica*’s growth characteristics of liking humid environments. The range of values shows that the precipitation does not exceed 300mm, indicating that extreme heavy rainfall may need to be taken into account when planting *A. dahurica*. In fact, in the traditional planting area of *A. dahurica* in the Sichuan Basin, the accumulation of rainy water in summer may pose a risk of waterlogging, requiring well drained soil and flood prevention measures; (2) The range of BIO_11 is −1.59 ∼ 9.13°C, which is consistent with the good cold resistance and preference for mild temperatures of *A. dahurica*, and indicates that it may not be suitable for climates that are too warm or too cold in winter; (3) The range of DEM values is 34.54 ∼ 692.17*m*, indicating that *A. dahurica* suitable for growth in low altitude (such as plains) to medium altitude (such as hills and mountains) areas. The increase in altitude in a region often has an impact on temperature (for every 100m increase in altitude, the temperature drops by 0.6°C), so there is an important connection between DEM and BIO_11. Within the range of values, when the altitude is high, *A. dahurica* will be in a lower temperature environment, and the average temperature in the coldest quarter may be close to the lower limit of the range, while at low altitudes it may be close to the upper limit. In addition, different altitudes can also form different terrains, such as windward slopes, leeward slopes, basins, etc., resulting in different climates, which is the main reason affecting precipitation. Considering three key variables, the ideal climate type for the growth environment of *A. dahurica* should be the monsoon climate zone, which includes the actual planting areas of *A. dahurica* such as Suining (subtropical humid monsoon climate), Yuzhou (warm temperate semi humid continental monsoon climate), Anguo (temperate monsoon climate), etc. The key variables selected by the model results of this study, although different from previous studies, are consistent with the actual situation and have high reliability.

### 4.2 Changes of suitable habitat for *A. dahurica*

In recent years, annual consumption has risen significantly, with sales exceeding 1000 tons in cities like Zhengzhou (Henan), Tengzhou (Shandong), Wuhan (Hubei), Chengdu (Sichuan), and Yulin (Guangxi). The demand for *A. dahurica* as a spice alone exceeds 8000 tons annually (data from www.zyctd.com). With the stimulation of the market, the planting area of *A. dahurica* in various regions is also increasing. For example, as of 2024, the city of Suining, known as the “hometown of Chinese white atractylodes”, will have a planting area of 25000 mu of white atractylodes, and is expected to increase to 30000 mu by 2025. But The planting area of traditional Chinese medicinal materials cannot determine the annual production capacity, and changes in environmental factors can lead to a decrease in yield per mu or even complete crop failure. For example, in low-lying areas such as Shangqiu in Henan and Bozhou in Anhui, due to abnormal weather, the growth period of *A. dahurica* shows a pattern of drought followed by flooding. These regions are on the edge of moderate suitability zones and adjacent to low suitability zones under the current climate background in this study. With future climate change, these areas will still be in moderate suitability zones, but adjacent areas may transition from low suitability zones to moderate suitability zones, which will have a positive impact on the cultivation of *A. dahurica*. At present, the emerging production area of Anhui, relying on China’s largest traditional Chinese medicine trading market, has become the largest main production area and distribution center of *A. dahurica* in the country, and is actively expanding its cultivation, which is consistent with the suitable area expansion direction predicted in this study, verifying the reliability of this study.

### 4.3 Environmental influence on the chemical composition of *A. dahurica*

Quality control is vital for ensuring both the therapeutic efficacy and economic value of medicinal plants. Studies indicate that coumarin compounds, particularly imperatorin and isoimperatorin, are key indicators of *A. dahurica* quality. Environmental variables influence the volatile composition and coumarin content of *A. dahurica* from different regions, affecting its pharmacological properties Sun et al. (2024); Zhang et al. (2019); Zhou et al. (2015). HPLC analysis based on the samples collected in the field, and the results showed that in the high suitable habitats, *A. dahurica* accumulated a relatively high content of coumarin overall. This indicates that under optimal growth conditions, plants can obtain more sufficient resources for metabolic synthesis, thereby improving the overall level of effective components. This makes *A. dahurica* from the high suitable habitats have better quality.

The constructed regression relationship model integrates the environmental data of the entire suitable area, revealing that different coumarins respond differently to environmental variables. Notably, BIO_11 is shown to influence the content of all coumarins examined in this research. Previous studies report that *A. dahurica* from southern Sichuan (low suitability area in this study) contains higher total coumarin, imperatorin, and isoimperatorin levels than samples from Yuzhou, Henan (high suitability area in this study) Zhang et al. (2024b). These findings align with predicted spatial distribution of coumarin content in this study. That is, although the total coumarin content in highly suitable areas is generally higher, the relative content of specific single components may not always be positively correlated with growth suitability. This may be due to: (1) Differences in the biosynthetic pathways and regulatory mechanisms of different coumarins; (2) In low suitable areas, some stress pressures may induce the activation of specific secondary metabolic pathways, thereby relatively increasing the content of some coumarins. For example, in plants such as tomatoes and tea trees, stress pressures often promote the accumulation of antioxidants such as flavonoids and phenols, which are considered to be adaptive strategies for plant self-protection Nakabayashi et al. (2014); Dixon and Paiva (1995). The combination of the two reveals the multi-level impact of environmental variables on the accumulation of chemical components in *A. dahurica*, that is, not only affecting the absolute content of the overall effective components, but also regulating the proportional distribution of each component. This finding provides a reference for the actual expansion of the planting area: when selecting the planting area, it is necessary to pay attention not only to the overall growth suitability, but also to consider the specific performance of each component under different environments, so as to achieve refined management of medicinal material quality. These results suggest that different environmental pressures may induce plants to initiate specific secondary metabolic responses, which have implications for further research on the ecological adaptation and metabolic regulation mechanisms of *A. dahurica* and other medicinal plants.

## 5 Conclusion

This study is the first to integrate MaxEnt with chemometrics to systematically explore the suitable habitat distribution of *A. dahurica* and its relationship with coumarin content and environmental variables. The key findings are as follows:

1. Environmental Influences: BIO_13, BIO_11, and DEM are the primary factors determining the distribution of *A. dahurica*.
2. Current Habitat Distribution: The most suitable habitats are concentrated in Sichuan, Henan, and Hebei.
3. Future Habitat Shifts: Under climate change scenarios, suitable regions will expand, with core distribution areas shifting toward Henan and Jiaozuo. Northern Hubei, central and southern Shaanxi, and parts of Shandong may transition from medium or low suitability to high suitability.
4. Chemical Composition Differences: HPLC and HCA analysis indicate that *A. dahurica* from highly suitable habitats contains higher total coumarin content than those from medium suitability regions. In addition, different coumarin compounds exhibit different content distributions, which can be used to classify *A. dahurica*, and this classification is consistent with habitat suitability.
5. Environmental Correlation: Multiple stepwise regression analysis reveals a significant correlation between five coumarin compounds and environmental variables, particularly BIO_11 and DEM, highlighting their impact on secondary metabolite accumulation.
6. Spatial Distribution of Coumarins: Different coumarin compounds exhibit varying spatial patterns, suggesting that habitat differences influence chemical composition.

Despite the promising insights provided by this study, several limitations should be acknowledged. First, the sample collection was primarily concentrated in moderately to highly suitable habitats, while low-suitability areas were underrepresented. This may limit the robustness of the regression models and the generalizability of chemical-environment relationships across the full ecological gradient of *A. dahurica*. Future studies should include more diverse sampling from marginal or low-suitability regions to enhance the comprehensiveness of spatial predictions.

Second, while the MaxEnt model offers high predictive performance based on presence-only data, it does not account for biotic interactions, land use changes, or anthropogenic disturbances, which may significantly influence the actual distribution of *A. dahurica*. Integrating land use data, socio-economic factors, and climate-resilient agricultural modeling would enhance future habitat suitability assessments.

Moreover, the current study focused primarily on five coumarin compounds. However, *A. dahurica* contains a wide spectrum of secondary metabolites, including volatile oils and polysaccharides, which also contribute to its pharmacological activity. Expanding the chemical spectrum and integrating metabolomics or transcriptomics approaches could provide a more holistic understanding of the plant’s adaptive responses.

Looking forward, this integrative framework combining species distribution modeling with chemometric analysis offers a valuable tool for ecological zoning, medicinal resource conservation, and precision cultivation. With the increasing impact of climate change, this approach could support dynamic planning of cultivation areas, ensuring both the quality and sustainability of *A. dahurica* resources. Additionally, the methodology can be extended to other traditional Chinese medicinal plants, paving the way for eco-pharmacological zoning and quality control strategies in herbal medicine production.

## Data Availability

The original contributions presented in the study are included in the article/**Supplementary Material**. Further inquiries can be directed to the corresponding authors.
