# Supplementary material for: Integrating MaxEnt with chemometrics to evaluate the impact of environmental variables on the coumarin content and the distribution of Angelica dahurica
Source: Front Plant Sci. 2025 Jul 7;16:1600491. doi: 10.3389/fpls.2025.1600491 (PMC12280906; doi:10.3389/fpls.2025.1600491)
Supplement: Supplementary file 1 [file DataSheet1.pdf]

Supplementary Material

1 SUPPLEMENTARY TABLES AND FIGURES

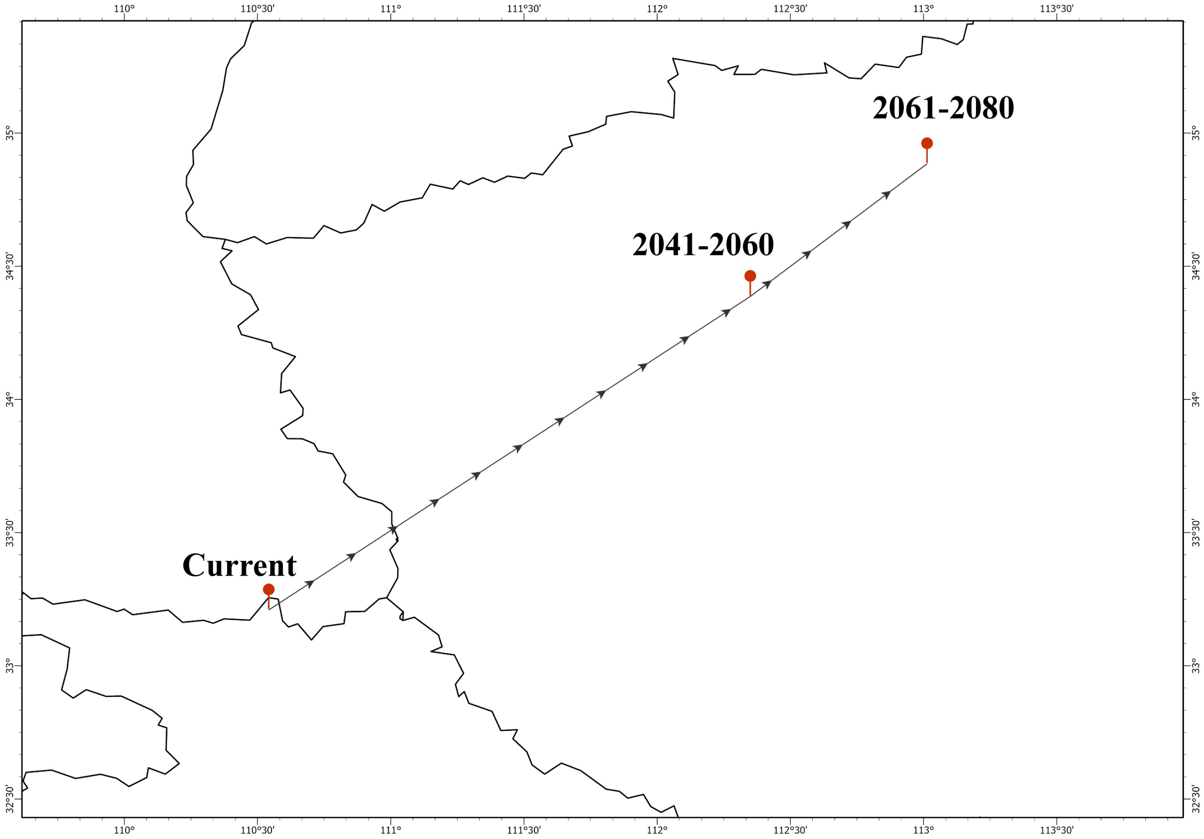

Figure S1: Migration routes of cores for suitable habitats under different time

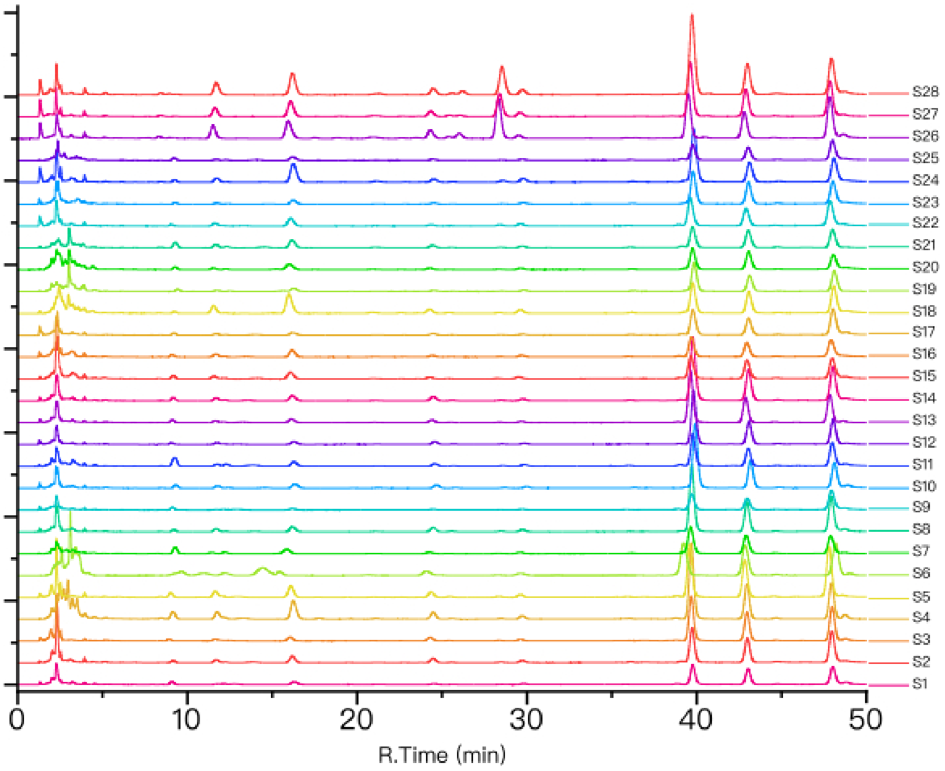

Figure S2: Sample chromatogram

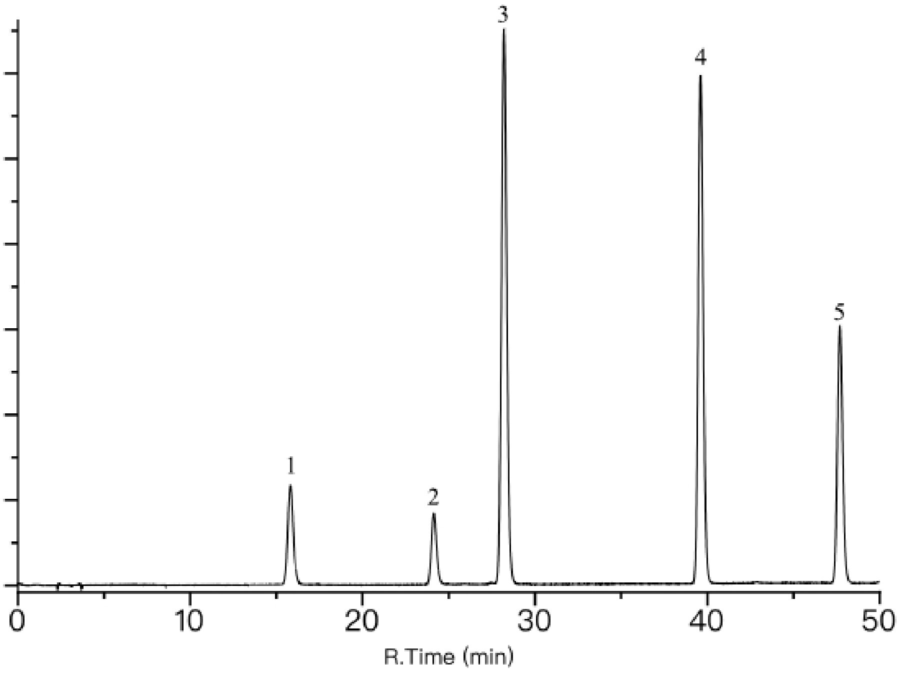

Figure S3: Standard product chromatogram;1-Oxypeucedanin hydrate,2-Bergapten,3-Oxypeucedanin,4-Imperatorin,5-Isoimperatorin

Table S1. Sample used for modeling

Species	Longitude	Latitude	Species	Longitude	Latitude	Species	Longitude	Latitude	Species	Longitude	Latitude	Species	Longitude	Latitude	Species	Longitude	Latitude
A. dahurica	123.0291667	50.62916667	A. dahurica	107.1958333	27.95416667	A. dahurica	110.9875	30.82916667	A. dahurica	116.1458333	39.75416667	A. dahurica	103.5541667	28.84583333	A. dahurica	112.1791667	34.5375
A. dahurica	123.7291667	50.59583333	A. dahurica	101.2875	27.9375	A. dahurica	115.4041667	30.7875	A. dahurica	113.6958333	39.7125	A. dahurica	108.7708333	28.84583333	A. dahurica	110.9041667	34.52083333
A. dahurica	124.1541667	50.42083333	A. dahurica	102.2708333	27.90416667	A. dahurica	107.2125	30.74583333	A. dahurica	114.7041667	39.3625	A. dahurica	104.6458333	28.75416667	A. dahurica	113.0541667	34.4625
A. dahurica	124.5125	48.4875	A. dahurica	106.4041667	27.79583333	A. dahurica	103.6791667	30.6375	A. dahurica	115.5041667	39.35416667	A. dahurica	102.5125	28.64583333	A. dahurica	111.6625	34.39583333
A. dahurica	124.5291667	48.4875	A. dahurica	107.4708333	27.75416667	A. dahurica	103.7041667	30.6125	A. dahurica	107.9708333	39.12083333	A. dahurica	105.7041667	28.59583333	A. dahurica	107.3791667	34.35416667
A. dahurica	126.4958333	48.24583333	A. dahurica	106.8375	27.54583333	A. dahurica	114.3125	30.59583333	A. dahurica	112.3291667	39.02083333	A. dahurica	104.9291667	28.5875	A. dahurica	107.7541667	34.27916667
A. dahurica	123.4625	48.12916667	A. dahurica	108.2291667	27.52083333	A. dahurica	104.0708333	30.57916667	A. dahurica	117.0791667	38.9625	A. dahurica	109.0125	28.45416667	A. dahurica	113.8208333	34.20416667
A. dahurica	122.7458333	48.02083333	A. dahurica	114.5625	27.39583333	A. dahurica	105.5958333	30.5375	A. dahurica	114.2041667	38.85416667	A. dahurica	102.4208333	28.3125	A. dahurica	105.1875	34.19583333
A. dahurica	125.9125	47.17916667	A. dahurica	114.6291667	27.39583333	A. dahurica	106.9375	30.3375	A. dahurica	114.9875	38.75416667	A. dahurica	105.2458333	28.3125	A. dahurica	113.6125	34.1875
A. dahurica	122.0458333	46.0875	A. dahurica	102.5458333	27.37916667	A. dahurica	107.3375	30.32916667	A. dahurica	113.2625	38.7375	A. dahurica	103.5791667	28.27083333	A. dahurica	108.2291667	34.17083333
A. dahurica	121.9208333	46.07083333	A. dahurica	107.9208333	26.9125	A. dahurica	111.0541667	30.17083333	A. dahurica	111.1375	38.47083333	A. dahurica	108.1291667	28.27083333	A. dahurica	113.4958333	34.14583333
A. dahurica	126.5375	45.8125	A. dahurica	104.2625	26.87916667	A. dahurica	102.9375	30.14583333	A. dahurica	115.5291667	38.2375	A. dahurica	113.0875	28.25416667	A. dahurica	112.0958333	34.1375
A. dahurica	126.9625	45.55416667	A. dahurica	106.5958333	26.84583333	A. dahurica	103.1125	30.07916667	A. dahurica	111.8041667	38.07083333	A. dahurica	104.7541667	31.7875	A. dahurica	108.6125	34.1125
A. dahurica	125.8458333	44.15416667	A. dahurica	113.2791667	26.7125	A. dahurica	102.7625	30.07083333	A. dahurica	112.5541667	37.87916667	A. dahurica	105.9375	31.7375	A. dahurica	103.2291667	34.0625
A. dahurica	126.5458333	43.84583333	A. dahurica	106.6375	26.65416667	A. dahurica	103.0375	30.02083333	A. dahurica	111.5208333	37.79583333	A. dahurica	103.8541667	31.67916667	A. dahurica	107.3291667	34.0625
A. dahurica	126.5541667	43.84583333	A. dahurica	102.5875	26.6375	A. dahurica	101.9625	30.00416667	A. dahurica	114.3958333	37.67083333	A. dahurica	114.8875	31.64583333	A. dahurica	111.0541667	34.0625
A. dahurica	125.3291667	43.82083333	A. dahurica	114.0041667	26.52916667	A. dahurica	104.1375	30.00416667	A. dahurica	112.1625	37.55416667	A. dahurica	99.99583333	31.62916667	A. dahurica	104.3958333	34.05416667
A. dahurica	122.2541667	43.65416667	A. dahurica	113.7791667	26.49583333	A. dahurica	108.1208333	30.00416667	A. dahurica	111.1541667	37.52083333	A. dahurica	107.9708333	31.60416667	A. dahurica	115.8375	33.99583333
A. dahurica	117.5541667	43.27083333	A. dahurica	112.9541667	25.40416667	A. dahurica	110.1791667	36.05416667	A. dahurica	112.5541667	37.42916667	A. dahurica	107.1125	31.5625	A. dahurica	115.9041667	33.9875
A. dahurica	118.8958333	42.2625	A. dahurica	103.0458333	25.34583333	A. dahurica	106.2458333	36.02083333	A. dahurica	114.5208333	37.29583333	A. dahurica	104.5708333	31.5375	A. dahurica	106.5208333	33.9125
A. dahurica	120.7791667	41.80416667	A. dahurica	106.5708333	24.35416667	A. dahurica	104.1208333	35.84583333	A. dahurica	104.0708333	37.1875	A. dahurica	103.5958333	31.47916667	A. dahurica	115.9041667	33.8125
A. dahurica	117.1875	41.77916667	A. dahurica	110.7875	29.9375	A. dahurica	111.7291667	35.74583333	A. dahurica	111.9208333	37.02916667	A. dahurica	104.4125	31.44583333	A. dahurica	113.3375	33.7375
A. dahurica	119.3541667	41.60416667	A. dahurica	107.7375	29.87083333	A. dahurica	106.1208333	35.62916667	A. dahurica	111.7875	36.85416667	A. dahurica	110.3458333	31.4375	A. dahurica	115.7541667	33.72083333
A. dahurica	120.4541667	41.57916667	A. dahurica	103.8541667	29.8375	A. dahurica	110.9875	35.6125	A. dahurica	112.7041667	36.7625	A. dahurica	109.5791667	31.40416667	A. dahurica	115.9958333	33.72083333
A. dahurica	117.7458333	41.32083333	A. dahurica	102.8708333	29.80416667	A. dahurica	109.8458333	35.5875	A. dahurica	114.2125	36.70416667	A. dahurica	107.7375	31.3625	A. dahurica	115.8875	33.67916667
A. dahurica	117.9708333	40.95416667	A. dahurica	107.3958333	29.7125	A. dahurica	116.9958333	35.5875	A. dahurica	117.1291667	36.65416667	A. dahurica	104.2291667	31.34583333	A. dahurica	107.9958333	33.52916667
A. dahurica	115.8375	40.92083333	A. dahurica	109.1458333	29.67083333	A. dahurica	106.3375	35.50416667	A. dahurica	101.7875	36.62083333	A. dahurica	114.6208333	31.29583333	A. dahurica	112.4375	33.49583333
A. dahurica	115.9791667	40.4625	A. dahurica	103.3625	29.57083333	A. dahurica	107.9375	35.50416667	A. dahurica	111.1041667	36.42083333	A. dahurica	107.5208333	31.20416667	A. dahurica	111.4791667	33.3125
A. dahurica	115.5208333	40.42083333	A. dahurica	104.6708333	29.5375	A. dahurica	112.4208333	35.49583333	A. dahurica	117.0958333	36.20416667	A. dahurica	108.3958333	31.1625	A. dahurica	107.5541667	33.22916667
A. dahurica	117.5041667	40.42083333	A. dahurica	108.7791667	29.5375	A. dahurica	113.8125	35.47083333	A. dahurica	112.2541667	36.15416667	A. dahurica	104.1708333	31.12916667	A. dahurica	106.6791667	33.1625
A. dahurica	118.9541667	40.4125	A. dahurica	106.6708333	29.50416667	A. dahurica	116.5958333	35.42083333	A. dahurica	111.5291667	36.09583333	A. dahurica	104.4041667	31.12916667	A. dahurica	107.3375	33.1625
A. dahurica	115.2125	40.3875	A. dahurica	105.6041667	29.4125	A. dahurica	109.1208333	35.40416667	A. dahurica	113.8291667	36.0875	A. dahurica	105.1041667	31.10416667	A. dahurica	111.8541667	33.05416667
A. dahurica	117.1875	40.27916667	A. dahurica	110.1708333	29.40416667	A. dahurica	115.5125	35.39583333	A. dahurica	103.8291667	36.0625	A. dahurica	107.8791667	31.0875	A. dahurica	106.9458333	33.00416667
A. dahurica	116.2375	40.22916667	A. dahurica	102.6625	29.35416667	A. dahurica	115.5875	35.39583333	A. dahurica	106.2958333	32.2375	A. dahurica	109.8875	31.07916667	A. dahurica	108.5125	32.89583333
A. dahurica	118.3208333	40.14583333	A. dahurica	108.1708333	29.29583333	A. dahurica	115.6125	35.39583333	A. dahurica	110.2375	32.22916667	A. dahurica	104.6875	31.0375	A. dahurica	108.5375	32.52916667
A. dahurica	116.1041667	39.94583333	A. dahurica	103.2708333	29.2375	A. dahurica	106.6625	35.22083333	A. dahurica	114.0958333	32.15416667	A. dahurica	103.6541667	30.99583333	A. dahurica	105.8541667	32.4375
A. dahurica	116.3958333	39.9125	A. dahurica	107.1041667	29.1625	A. dahurica	113.8125	35.19583333	A. dahurica	108.0375	32.0875	A. dahurica	101.1291667	30.9875	A. dahurica	104.5375	32.4125
A. dahurica	116.4125	39.9125	A. dahurica	110.4875	29.12083333	A. dahurica	111.0125	35.02916667	A. dahurica	108.1708333	31.97083333	A. dahurica	103.9541667	30.97083333	A. dahurica	106.8375	32.35416667
A. dahurica	115.6208333	39.8625	A. dahurica	102.7791667	28.9625	A. dahurica	110.4541667	34.87083333	A. dahurica	104.3625	31.9625	A. dahurica	105.3958333	30.87916667	A. dahurica	108.9125	32.3125
A. dahurica	116.2375	39.85416667	A. dahurica	105.4458333	28.87916667	A. dahurica	105.7291667	34.5875	A. dahurica	108.6708333	31.95416667	A. dahurica	106.0958333	30.8375	A. dahurica	105.5291667	32.29583333
A. dahurica	114.5958333	39.84583333	A. dahurica	104.9791667	28.85416667	A. dahurica	110.0958333	34.57083333	A. dahurica	107.2541667	31.92083333	A. dahurica	106.7625	31.87083333	A. dahurica	98.5875	31.8125

Table S2. Correlation analysis between environmental variables

	ADD_PROP	AWC_CLASS	BIO_1	BIO_10	BIO_11	BIO_12	BIO_13	BIO_14	BIO_15	BIO_16
ADD_PROP	1	NA	NA	NA	NA	NA	NA	NA	NA	NA
AWC_CLASS	NA	1	0.155849975	0.021511857	0.10214774	0.171981798	0.172132593	0.15182782	0.225701407	0.166643852
BIO_1	NA	0.155849975	1	0.225015116	0.983905475	0.906474034	0.801117727	0.630767281	0.469931601	0.885708063
BIO_10	NA	0.021511857	0.225015116	1	0.168346673	0.050931008	0.001973252	0.357324668	0.166356417	0.050887384
BIO_11	NA	0.10214774	0.983905475	0.168346673	1	0.893682299	0.772274089	0.587317851	0.523210035	0.870441359
BIO_12	NA	0.171981798	0.906474034	0.050931008	0.893682299	1	0.764735175	0.757304508	0.607858163	0.828826383
BIO_13	NA	0.172132593	0.801117727	0.001973252	0.772274089	0.764735175	1	0.498662045	0.187205699	0.957593895
BIO_14	NA	0.15182782	0.630767281	0.357324668	0.587317851	0.757304508	0.498662045	1	0.591974503	0.490767882
BIO_15	NA	0.225701407	0.469931601	0.166356417	0.523210035	0.607858163	0.187205699	0.591974503	1	0.240175163
BIO_16	NA	0.166643852	0.885708063	0.050887384	0.870441359	0.828826383	0.957593895	0.490767882	0.240175163	1
BIO_17	NA	0.166881588	0.611139401	0.381678738	0.564597988	0.730896723	0.501755882	0.983599734	0.556019272	0.48623802
BIO_19	NA	0.166881588	0.611139401	0.381678738	0.564597988	0.730896723	0.501755882	0.983599734	0.556019272	0.48623802
BIO_2	NA	0.204206035	0.926073873	0.060562935	0.90633904	0.904071364	0.729434027	0.692293256	0.467607792	0.84685281
BIO_3	NA	0.354654234	0.703326772	0.136669816	0.675570721	0.688590981	0.441645455	0.304197076	0.133391695	0.584642372
BIO_4	NA	0.021494196	0.851718105	0.169632648	0.895725107	0.799233937	0.747029267	0.442217529	0.464518248	0.868370706
BIO_5	NA	0.232723754	0.556114354	0.364756688	0.553956865	0.622445668	0.748924623	0.467360711	0.103380581	0.764198151
BIO_6	NA	0.18302137	0.984050881	0.186056797	0.972309815	0.918051919	0.784056819	0.673339477	0.483311194	0.875048796
BIO_8	NA	0.021500078	0.163811507	0.521141496	0.21271354	0.267323483	0.363646431	0.000828349	0.327933458	0.356345124
BIO_9	NA	0.102153335	0.982864432	0.16693147	0.999068954	0.896249724	0.779220874	0.59524911	0.528165609	0.869941417
DEM	NA	0.064482588	0.735062381	0.352735157	0.762384459	0.806128602	0.71132083	0.395511408	0.521529792	0.811340435
REF_DEPTH	NA	NA	NA	NA	NA	NA	NA	NA	NA	NA
SU_SYM90	NA	0.077732334	0.304224581	0.515643453	0.353433945	0.342688366	0.296415696	0.071534151	0.151736498	0.345364815
T_CACO3	NA	0.298416141	0.487533691	0.123941117	0.460531144	0.384578438	0.574726249	0.302563471	0.071910661	0.581046618
T_CEC_CLAY	NA	0.065386754	0.458740028	0.388441072	0.480895858	0.551594605	0.32577115	0.206014871	0.298763895	0.428230158
T_CEC_SOIL	NA	0.339748242	0.243895605	0.1362455	0.273127109	0.262602501	0.32002373	0.139173394	0.063439517	0.311943642
T_GRAVEL	NA	0.352214044	0.419053812	0.2115267	0.375543921	0.402208318	0.549910523	0.446962197	0.075703493	0.505827321
T_OC	NA	0.111719431	0.405713898	0.182388212	0.375792956	0.550218134	0.322726312	0.397389074	0.348733981	0.353237581
T_PH_H2O	NA	0.30616527	0.173086431	0.2617488	0.162673785	0.046391697	0.293822066	0.131610284	0.089531165	0.254711266
T_SAND	NA	0.370524937	0.412688477	0.201438566	0.364446238	0.458487699	0.569571925	0.51458932	0.150075457	0.486504091
T_SILT	NA	0.370524937	0.419124598	0.264068859	0.382318867	0.364271057	0.498153293	0.376313034	0.030791822	0.478179243
T_TEXTURE	NA	0.449431893	0.402425678	0.013665035	0.383528999	0.479807422	0.49729469	0.438363796	0.146140727	0.459165507

	BIO_17	BIO_19	BIO_2	BIO_3	BIO_4	BIO_5	BIO_6	BIO_8	BIO_9	DEM	REF_DEPTH
ADD_PROP	NA	NA	NA	NA	NA	NA	NA	NA	NA	NA	NA
AWC_CLASS	0.166881588	0.166881588	0.204206035	0.354654234	0.021494196	0.232723754	0.18302137	0.021500078	0.102153335	0.064482588	NA
BIO_1	0.611139401	0.611139401	0.926073873	0.703326772	0.851718105	0.556114354	0.984050881	0.163811507	0.982864432	0.735062381	NA
BIO_10	0.381678738	0.381678738	0.060562935	0.136669816	0.169632648	0.364756688	0.186056797	0.521141496	0.16693147	0.352735157	NA
BIO_11	0.564597988	0.564597988	0.90633904	0.675570721	0.895725107	0.553956865	0.972309815	0.21271354	0.999068954	0.762384459	NA
BIO_12	0.730896723	0.730896723	0.904071364	0.688590981	0.799233937	0.622445668	0.918051919	0.267323483	0.896249724	0.806128602	NA
BIO_13	0.501755882	0.501755882	0.729434027	0.441645455	0.747029267	0.748924623	0.784056819	0.363646431	0.779220874	0.71132083	NA
BIO_14	0.983599734	0.983599734	0.692293256	0.304197076	0.442217529	0.467360711	0.673339477	0.000828349	0.59524911	0.395511408	NA
BIO_15	0.556019272	0.556019272	0.467607792	0.133391695	0.464518248	0.103380581	0.483311194	0.327933458	0.528165609	0.521529792	NA
BIO_16	0.48623802	0.48623802	0.84685281	0.584642372	0.868370706	0.764198151	0.875048796	0.356345124	0.869941417	0.811340435	NA
BIO_17	1	1	0.662520204	0.248506847	0.407345144	0.481983173	0.643770934	0.014638175	0.57230646	0.345519886	NA
BIO_19	1	1	0.662520204	0.248506847	0.407345144	0.481983173	0.643770934	0.014638175	0.57230646	0.345519886	NA
BIO_2	0.662520204	0.662520204	1	0.749357117	0.858346557	0.656102983	0.950672789	0.192042474	0.902775606	0.784483231	NA
BIO_3	0.248506847	0.248506847	0.749357117	1	0.590983696	0.385506915	0.72048779	0.031084114	0.672213808	0.568661779	NA
BIO_4	0.407345144	0.407345144	0.858346557	0.590983696	1	0.664883711	0.867698364	0.434411443	0.893256101	0.886858518	NA
BIO_5	0.481983173	0.481983173	0.656102983	0.385506915	0.664883711	1	0.568511299	0.438857354	0.554538545	0.650666904	NA
BIO_6	0.643770934	0.643770934	0.950672789	0.72048779	0.867698364	0.568511299	1	0.158818221	0.971266335	0.756221705	NA
BIO_8	0.014638175	0.014638175	0.192042474	0.031084114	0.434411443	0.438857354	0.158818221	1	0.216010521	0.608767056	NA
BIO_9	0.57230646	0.57230646	0.902775606	0.672213808	0.893256101	0.554538545	0.971266335	0.216010521	1	0.758046971	NA
DEM	0.345519886	0.345519886	0.784483231	0.568661779	0.886858518	0.650666904	0.756221705	0.608767056	0.758046971	1	NA
REF_DEPTH	NA	NA	NA	NA	NA	NA	NA	NA	NA	NA	1
SU_SYM90	0.102732198	0.102732198	0.273397058	0.398993148	0.451792751	0.28992337	0.314012549	0.303200161	0.360523499	0.508210314	NA
T_CACO3	0.304324913	0.304324913	0.473102375	0.249929326	0.387216033	0.534223779	0.488899678	0.011593771	0.45278754	0.342541588	NA
T_CEC_CLAY	0.174960045	0.174960045	0.45893685	0.538133932	0.545013169	0.338947234	0.462051446	0.225578668	0.483975142	0.577412161	NA
T_CEC_SOIL	0.167233295	0.167233295	0.207782552	0.117498956	0.235890592	0.344017085	0.237925484	0.105755677	0.280510707	0.224397342	NA
T_GRAVEL	0.449124343	0.449124343	0.433487952	0.233205551	0.281617276	0.489347124	0.43602129	0.010312726	0.373658355	0.235783027	NA
T_OC	0.349346198	0.349346198	0.432748645	0.457911738	0.41468799	0.264889374	0.415415913	0.237551864	0.384674381	0.482773518	NA
T_PH_H2O	0.14115296	0.14115296	0.153749201	0.118079909	0.106109823	0.232520256	0.174245635	0.040472457	0.154323998	0.07219458	NA
T_SAND	0.497091056	0.497091056	0.359959173	0.11339647	0.283824041	0.368003047	0.412478449	0.088289215	0.373958083	0.290925189	NA
T_SILT	0.35114281	0.35114281	0.36994571	0.128486411	0.326430933	0.29592228	0.432040887	0.043228975	0.375068245	0.292700477	NA
T_TEXTURE	0.449691049	0.449691049	0.358709057	0.214328006	0.300826774	0.416201675	0.394659491	0.06873071	0.394995952	0.297136554	NA

	SU_SYM90	T_CACO3	T_CEC_CLAY	T_CEC_SOIL	T_GRAVEL	T_OC	T_PH_H2O	T_SAND	T_SILT	T_TEXTURE
ADD_PROP	NA	NA	NA	NA	NA	NA	NA	NA	NA	NA
AWC_CLASS	0.077732334	0.298416141	0.065386754	0.339748242	0.352214044	0.111719431	0.30616527	0.370524937	0.370524937	0.449431893
BIO_1	0.304224581	0.487533691	0.458740028	0.243895605	0.419053812	0.405713898	0.173086431	0.412688477	0.419124598	0.402425678
BIO_10	0.515643453	0.123941117	0.388441072	0.1362455	0.2115267	0.182388212	0.2617488	0.201438566	0.264068859	0.013665035
BIO_11	0.353433945	0.460531144	0.480895858	0.273127109	0.375543921	0.375792956	0.162673785	0.364446238	0.382318867	0.383528999
BIO_12	0.342688366	0.384578438	0.551594605	0.262602501	0.402208318	0.550218134	0.046391697	0.458487699	0.364271057	0.479807422
BIO_13	0.296415696	0.574726249	0.32577115	0.32002373	0.549910523	0.322726312	0.293822066	0.569571925	0.498153293	0.49729469
BIO_14	0.071534151	0.302563471	0.206014871	0.139173394	0.446962197	0.397389074	0.131610284	0.51458932	0.376313034	0.438363796
BIO_15	0.151736498	0.071910661	0.298763895	0.063439517	0.075703493	0.348733981	0.089531165	0.150075457	0.030791822	0.146140727
BIO_16	0.345364815	0.581046618	0.428230158	0.311943642	0.505827321	0.353237581	0.254711266	0.486504091	0.478179243	0.459165507
BIO_17	0.102732198	0.304324913	0.174960045	0.167233295	0.449124343	0.349346198	0.14115296	0.497091056	0.35114281	0.449691049
BIO_19	0.102732198	0.304324913	0.174960045	0.167233295	0.449124343	0.349346198	0.14115296	0.497091056	0.35114281	0.449691049
BIO_2	0.273397058	0.473102375	0.45893685	0.207782552	0.433487952	0.432748645	0.153749201	0.359959173	0.36994571	0.358709057
BIO_3	0.398993148	0.249929326	0.538133932	0.117498956	0.233205551	0.457911738	0.118079909	0.11339647	0.128486411	0.214328006
BIO_4	0.451792751	0.387216033	0.545013169	0.235890592	0.281617276	0.41468799	0.106109823	0.283824041	0.326430933	0.300826774
BIO_5	0.28992337	0.534223779	0.338947234	0.344017085	0.489347124	0.264889374	0.232520256	0.368003047	0.29592228	0.416201675
BIO_6	0.314012549	0.488899678	0.462051446	0.237925484	0.43602129	0.415415913	0.174245635	0.412478449	0.432040887	0.394659491
BIO_8	0.303200161	0.011593771	0.225578668	0.105755677	0.010312726	0.237551864	0.040472457	0.088289215	0.043228975	0.06873071
BIO_9	0.360523499	0.45278754	0.483975142	0.280510707	0.373658355	0.384674381	0.154323998	0.373958083	0.375068245	0.394995952
DEM	0.508210314	0.342541588	0.577412161	0.224397342	0.235783027	0.482773518	0.07219458	0.290925189	0.292700477	0.297136554
REF_DEPTH	NA	NA	NA	NA	NA	NA	NA	NA	NA	NA
SU_SYM90	1	0.067554961	0.805621501	0.382094334	0.289017301	0.310616822	0.320544893	0.168759762	0.261738137	0.151527911
T_CACO3	0.067554961	1	0.078392175	0.516169602	0.714193078	0.328041224	0.828757596	0.261649572	0.326006467	0.326679521
T_CEC_CLAY	0.805621501	0.078392175	1	0.331867723	0.283066091	0.682822543	0.503471164	0.168766877	0.038928893	0.44035435
T_CEC_SOIL	0.382094334	0.516169602	0.331867723	1	0.199990409	0.229009314	0.320682623	0.198011768	0.045599281	0.739618187
T_GRAVEL	0.289017301	0.714193078	0.283066091	0.199990409	1	0.13280339	0.660720735	0.434883151	0.558745873	0.308244272
T_OC	0.310616822	0.328041224	0.682822543	0.229009314	0.13280339	1	0.561799142	0.480480341	0.331195297	0.29527003
T_PH_H2O	0.320544893	0.828757596	0.503471164	0.320682623	0.660720735	0.561799142	1	0.186367779	0.314068903	0.109164571
T_SAND	0.168759762	0.261649572	0.168766877	0.198011768	0.434883151	0.480480341	0.186367779	1	0.863861386	0.751685774
T_SILT	0.261738137	0.326006467	0.038928893	0.045599281	0.558745873	0.331195297	0.314068903	0.863861386	1	0.538244135
T_TEXTURE	0.151527911	0.326679521	0.44035435	0.739618187	0.308244272	0.29527003	0.109164571	0.751685774	0.538244135	1
